# Fast Simulation of Mechanical Heterogeneity in the Electrically Asynchronous Heart Using the MultiPatch Module

**DOI:** 10.1371/journal.pcbi.1004284

**Published:** 2015-07-23

**Authors:** John Walmsley, Theo Arts, Nicolas Derval, Pierre Bordachar, Hubert Cochet, Sylvain Ploux, Frits W. Prinzen, Tammo Delhaas, Joost Lumens

**Affiliations:** 1 Department of Biomedical Engineering, Cardiovascular Research Institute Maastricht (CARIM), Maastricht University, Maastricht, The Netherlands; 2 Hôpital Cardiologique du Haut-Lévêque, IHU-LIRYC, CHU de Bordeaux, Bordeaux, France; 3 Department of Physiology, Cardiovascular Research Institute Maastricht (CARIM), Maastricht University, Maastricht, The Netherlands; University of California, San Diego, UNITED STATES

## Abstract

Cardiac electrical asynchrony occurs as a result of cardiac pacing or conduction disorders such as left bundle-branch block (LBBB). Electrically asynchronous activation causes myocardial contraction heterogeneity that can be detrimental for cardiac function. Computational models provide a tool for understanding pathological consequences of dyssynchronous contraction. Simulations of mechanical dyssynchrony within the heart are typically performed using the finite element method, whose computational intensity may present an obstacle to clinical deployment of patient-specific models. We present an alternative based on the CircAdapt lumped-parameter model of the heart and circulatory system, called the MultiPatch module. Cardiac walls are subdivided into an arbitrary number of patches of homogeneous tissue. Tissue properties and activation time can differ between patches. All patches within a wall share a common wall tension and curvature. Consequently, spatial location within the wall is not required to calculate deformation in a patch. We test the hypothesis that activation time is more important than tissue location for determining mechanical deformation in asynchronous hearts. We perform simulations representing an experimental study of myocardial deformation induced by ventricular pacing, and a patient with LBBB and heart failure using endocardial recordings of electrical activation, wall volumes, and end-diastolic volumes. Direct comparison between simulated and experimental strain patterns shows both qualitative and quantitative agreement between model fibre strain and experimental circumferential strain in terms of shortening and rebound stretch during ejection. Local myofibre strain in the patient simulation shows qualitative agreement with circumferential strain patterns observed in the patient using tagged MRI. We conclude that the MultiPatch module produces realistic regional deformation patterns in the asynchronous heart and that activation time is more important than tissue location within a wall for determining myocardial deformation. The CircAdapt model is therefore capable of fast and realistic simulations of dyssynchronous myocardial deformation embedded within the closed-loop cardiovascular system.

## Author Summary

Under normal conditions, the electrical activation of the heart is almost synchronous, leading to uniform contraction. Due to either pathology or electrical pacing, the heart can be activated asynchronously. The result is discoordinated contraction and a reduction in the ability to pump blood. There is considerable interest in using computer simulations to understand how asynchronous electrical activation affects cardiac deformation, and how pathologies of the cardiac conduction system can be treated by pacing the heart. We present the MultiPatch module for simulating the effects of asynchronous electrical activation on cardiac contraction in the relatively simple CircAdapt model of the heart and circulation. We quantitatively compare model simulations to deformation patterns recorded during an experimental study of pacing-induced electrical asynchrony. We then demonstrate a ‘patient-specific’ simulation of deformation in a patient with a conduction disorder called left bundle-branch block. We use timings from endocardial mapping of electrical activation in a patient as an input for the model, and compare the resulting simulated deformation patterns to tagged magnetic resonance imaging recordings from the same patient. The model qualitatively reproduces deformation as observed in the patient. We conclude that the MultiPatch module makes CircAdapt appropriate for simulation of dyssynchronous heart failure in patients.

## Introduction

The ventricles of the heart undergo rapid electrical activation under normal conditions through the cardiac conduction system, leading to a near-synchronous mechanical contraction of the ventricles [1]. Ventricular electrical asynchrony may be induced either by ventricular pacing or through disorders of the cardiac conduction system such as left bundle-branch block (LBBB) [2]. Electrically asynchronous ventricular activation results in reduced pump function, due to dis-coordinated contraction and relaxation of the inter-ventricular septum and left ventricular (LV) free wall [3,4]. Ventricular deformation recorded during tagged MRI [5] or echocardiography [6] may contain information on both the electrical activation pattern causing mechanical dyssynchrony, and the health of ventricular tissue [7]. However, confusion remains due to conflicting data over the usefulness of mechanical dyssynchrony as a marker for potential response to treatment of dyssynchronous heart failure with cardiac resynchronization therapy (CRT) [7–9].

Theoretical studies using computational models offer the ability to mechanistically link ventricular electrical activation and tissue condition to both mechanical deformation and haemodynamic changes. Computational models are beginning to provide insights into dyssynchronous heart failure and its treatment with CRT [7,10–17]. There is considerable interest in producing ‘patient-specific’ models with the ultimate goal of predicting response to treatment of dyssynchronous heart failure with CRT [18–21].

The Finite Element (FE) method is the standard method to simulate time-dependent cardiac mechanics with inhomogeneous mechanical behavior of the myocardium. Common outputs are spatial distributions of stress and strain, and changes in cardiac geometry during ventricular systole. Patient-specific modeling using the FE method requires a considerable amount of input data such as a cardiac geometry from the patient and spatial distribution of mechanical properties within the myocardium, including fibre orientation, stiffness, and contractility [22]. Many of these ventricular properties are difficult to obtain in a clinical setting, requiring the use of generalised data instead based on anatomical atlases [23]. Furthermore, simulations using the FE method require considerable computational resources preventing availability of calculated results during clinical measurement protocols.

**Fig 1:**
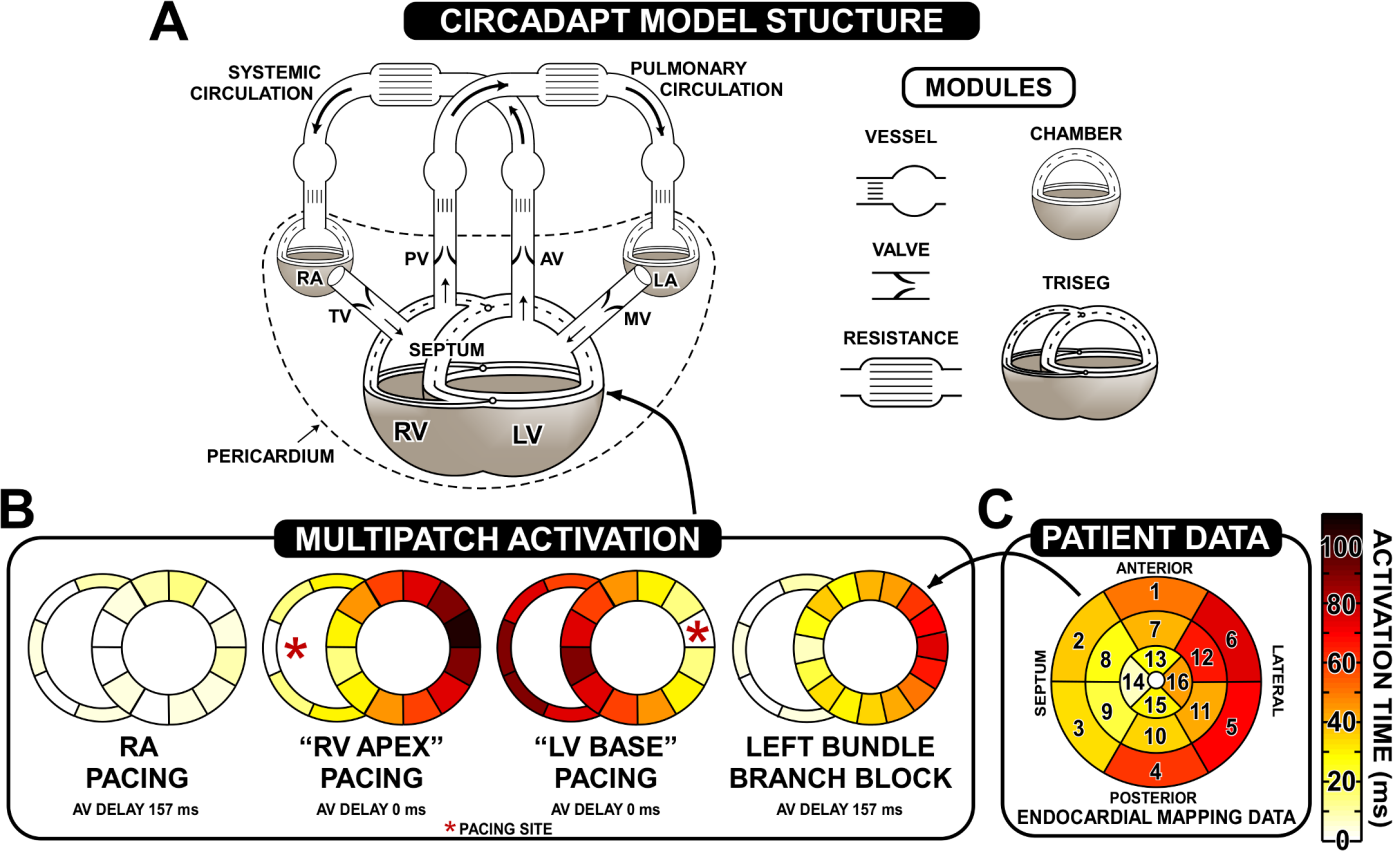
Simulation protocol. Panel A shows a schematic representation of the CircAdapt model, adapted from Lumens *et al* (2009) [25]. The TriSeg module representing mechanical interaction between the left and right ventricles, the chamber module representing the left and right atria, the cardiac valves, major vessels, and systemic and pulmonary resistances are shown. Panel B shows activation patterns used for the simulation of RA pacing, RV apex pacing, and LV base pacing, together with the atrio-ventricular (AV) delay. The activation map for the patient specific simulation of LBBB is shown, together with the endocardial mapping data recorded from the endocardium plotted on a standard AHA segmentation without the apical segment in panel C. The colour bar denotes time of activation in ms, and is common to all activation maps. The asterisk (*) indicates the approximate location of pacing in the model for reference.

In an attempt to reduce the input information required, and to focus on clinically measurable output data, the CircAdapt model of the heart and circulation has been developed (Fig 1A) [24]. CircAdapt allows rapid simulation of cardiac pump function and cardiovascular system dynamics for both research and educational purposes (www.circadapt.org). The model uses a highly simplified ventricular geometry where the cardiac walls are represented by thick-walled spherical shells consisting of myofibres. In the original CircAdapt model, mechanical properties and load were assumed to be evenly distributed within each wall. In the present study we introduce the MultiPatch module, allowing simulation of the effects of an uneven distribution of mechanical properties in the cardiac walls within the CircAdapt framework. Advantages of this approach over FE implementations include an extremely low computational effort, and a smooth haemodynamic incorporation of the heart within the entire circulation. Thus, real-time simulation of cardiac mechanics and haemodynamics is feasible using CircAdapt, widening the possibilities for patient-specific modeling in a clinical setting.

The MultiPatch module subdivides a cardiac wall into a finite number of patches. Each patch has its own stress-strain relation for the constituent myofibers. Although cardiac geometry is known to be complicated, we postulated that a cardiac wall may be considered simply spherical to estimate mechanical load in the tissue. The hypothesis used is that each patch can be considered part of a spherical wall. Furthermore, in this wall, the two-dimensional tensor, representing wall tension, is considered isotropic. An important consequence of this hypothesis is that wall tension is homogeneous in each wall, according to Laplace’s law. The location of a patch within a wall is therefore not relevant for the calculation of stress and strain in the patch, since all patches in a wall behave as if in series and experience the same wall tension. Myofibre stress is not necessarily distributed homogeneously as the thickness of the patches may differ. For example, when simulating mechanical effects of asynchronous activation, the location in the wall determines timing of activation, which then determines fibre stress and strain in the patch. After performing the calculations fibre mechanics are known locally, given the activation time of the tissue. Consequently, computational effort is reduced tremendously relative to the FE method.

In the present study, we quantitatively assessed the effectiveness of the MultiPatch module for simulating mechanical deformation during asynchronous ventricular activation. For that purpose, we simulated experiments in a well-known animal study into the effects of pacing-induced electrical dyssynchrony [4]. We directly compare strain, stress-strain loops, and haemodynamic response calculated by the model to experimental recordings to investigate the hypothesis underpinning the MultiPatch module, that activation time of ventricular tissue is more important than tissue location for determining myocardial deformation in the asynchronous heart. Furthermore, we show a proof-of-concept simulation of ventricular mechanical deformation in a patient with LBBB and heart failure. We use recordings of endocardial electrical activation, and the patient’s stroke volume and ejection fraction as inputs to our model, and compare the simulated deformation patterns of the left ventricular and septal walls to those recorded in the patient using tagged MRI.

## Results

### The CircAdapt Model

The CircAdapt model, as shown schematically in Fig 1A, simulates the heart when incorporated in the whole circulation to provide realistic loading. The heart consists of five walls, *i*.*e*., the left and right atrial walls, the left and right ventricular wall, and the interventricular septum. Cavity pressures are calculated from cavity volumes as follows. Cavity volumes determine wall area. Wall areas determine strain of the myofibres in the wall. A model of myofibre mechanics is used to calculate myofibre stress from myofibre strain. Myofibre stress determines wall tension in each cardiac wall. Using the TriSeg module [25], the mechanical equilibrium between the three ventricular walls is used to calculate their shape when encapsulating the two ventricular cavities. Transmural pressure is calculated from wall tension and curvature for each wall using Laplace’s law. Cavity pressures are found by adding the transmural pressures to the intra-pericardial pressure surrounding the myocardial walls.

### The MultiPatch Module

In the basic CircAdapt model, wall area determines wall tension. In a cardiac wall, consisting of *n* different patches, the total area of the patches adds up to the given total wall area. The wall area is the area of the surface that divides the wall volume in half (see Materials and Methods). The MultiPatch module is designed 1) to calculate wall tension; and 2) to determine the area assigned to the different patches. To solve for the unknown areas of *n* patches we use one equation, saying that the sum of all patch areas equals the given total wall area. Furthermore we use a set of *n*-1 equations, saying that wall tension in all patches is the same. Thus, in total, *n* equations are used to solve the area of *n* patches. From the area and the known constitutive equations of each patch, myofibre strains and stresses are calculated. For each patch, the resulting wall tension is calculated. Because of the applied set of equations wall tension is general to all patches. Thus, as in the regular wall, the MultiPatch module determines wall tension from wall area. The mechanical load of the myofibres in each patch is an important side result of these calculations. Details of the mathematical derivation are presented in the methods section.

### Simulation Protocols

#### Simulation of a paced canine model of dyssynchrony

We simulated electrical asynchrony as induced in a paced canine model by Prinzen *et al* [4]. The LV free wall and septum were divided into 12 patches. We considered three pacing modes: right atrial (RA) pacing, RV apex pacing, and LV base pacing. In RA pacing, we assume a relatively synchronous ventricular activation due to activation of the His-Purkinje system. We assume that during ventricular pacing activation time of tissue increases with distance from the pacing site. In the MultiPatch model, this is represented by gradually increasing the proportion of patches in the wall that are activated. When simulating LV basal pacing, the earliest activated ventricular patch is in the left ventricular free wall, representing the pacing site. Following the first activation of a patch within a wall, two more patches become activated every 15 ms until every patch in the wall is activated, representing propagation of excitation through the myocardium away from the stimulus site. Septal and RV patches are then sequentially activated following the activation of all left ventricular patches, resulting in a delayed activation of the septum consistent with experimental recordings in LV-paced dogs [26]. The activation pattern is reversed to represent RV apex pacing, with an RV segment activated first, followed by a septal segment. Activation patterns are represented diagrammatically in Fig 1B. As in the study of Prinzen *et al* [4], we set the AV delay to 0 ms during both ventricular pacing simulations, whereas in RA pacing we used an AV delay of 157 ms.

#### Patient-specific simulation of mechanical deformation with left bundle-branch block

To simulate cardiac mechanics in the patient with LBBB and heart failure, patches are assigned a time of onset of activation in Fig 1B corresponding to one segment in the standard AHA segmentation of the LV [27]. The endocardial mapping data is shown in Fig 1C. The LV and septum in the model were split into 16 segments and each was assigned an activation time corresponding to one of the AHA segments. Because we consider patches to be mechanically coupled in series, the order in which patches are placed within a wall is not significant. The resulting activation pattern used in the simulations is shown in Fig 1B. The patient’s heart rate of 76 beats per minute, and stroke volume of 63 mL were used as inputs to the model. Heart rate and stroke volume determined systemic flow. The systemic circulation was adapted to this flow using the CircAdapt adaptation protocols [28]. Septal and LV free wall volumes as derived from the MRI recordings were then used to determine the LV wall volume in the model. LV failure was induced by reducing the ability of the septum and LV free wall to generate active stress through reduction of the parameter σ_fAct_ (see online supplement, ‘The CircAdapt sarcomere contraction model’) to 60% of its original value. Dilation was reproduced by increasing the reference mid-wall area A_pRef_ (see Materials and Methods) by 35% in all LV free wall and septal patches, to give an LV ejection fraction and end-diastolic volume close to the patient’s values of 24% and 266 mL, respectively. Because no mitral regurgitation was observed in the patient we prevented mitral regurgitation from occurring in these simulations. All simulations shown are at a haemodynamic steady state.

### Paced Canine Experiments

#### Simulated pump function decreases with pacing

In the baseline RA pacing simulations, we recorded a peak systolic pressure of 117 mmHg, and an end-diastolic pressure of 11 mmHg. The stroke volume was 71 mL and the end-diastolic volume was 128 mL. During both RV apex pacing and LV basal pacing, peak systolic pressure decreased compared to RA pacing to values of 93 mmHg and to 95 mmHg, respectively. End diastolic pressure decreased slightly in the simulations to 7 mmHg during RV apex pacing and to 8 mmHg during LV base pacing, but stayed constant in the experiments (Table 1). The stroke volume in ventricular pacing simulations decreased to 55 mL for RV apex pacing, and to 57 mL for LV basal pacing. The end diastolic volume also decreased slightly, to 106 mL with RV apex pacing and to 110 mL with LV base pacing. Further haemodynamic information is provided in Table 1.

**Table 1:** Haemodynamic parameters from pacing simulations.

Parameter	RA Pacing		RV Apex Pacing		LV Base pacing	
	Experiment	Simulation	Experiment	Simulation	Experiment	Simulation
LV Stroke volume	19.9 ± 13.9 mL	71 mL	14.8 ± 6 mL *	55 mL	17.4 ± 9.5 mL	57 mL
RV stroke volume	-	71 mL	-	55 mL	-	57 mL
LV peak systolic pressure	105 ± 12 mmHg	117mmHg	96 ± 20 mmHg *	93 mmHg	103 ± 20 mmHg	95 mmHg
RV peak systolic pressure	-	34 mmHg	-	32 mmHg	-	33 mmHg
LV End diastolic volume	-	128 mL	-	106 mL	-	110 mL
RV end diastolic volume	-	108 mL	-	98 mL	-	91 mL
LV end diastolic pressure	10 ± 2 mmHg	11 mmHg	9 ± 2 mmHg	7 mmHg	10 ± 2 mmHg	8 mmHg
RV end diastolic pressure	-	4 mmHg	-	3 mmHg	-	3 mmHg

* denotes statistical significance relative to RA pacing

#### Simulated and experimental strain patterns are consistent

**Fig 2:**
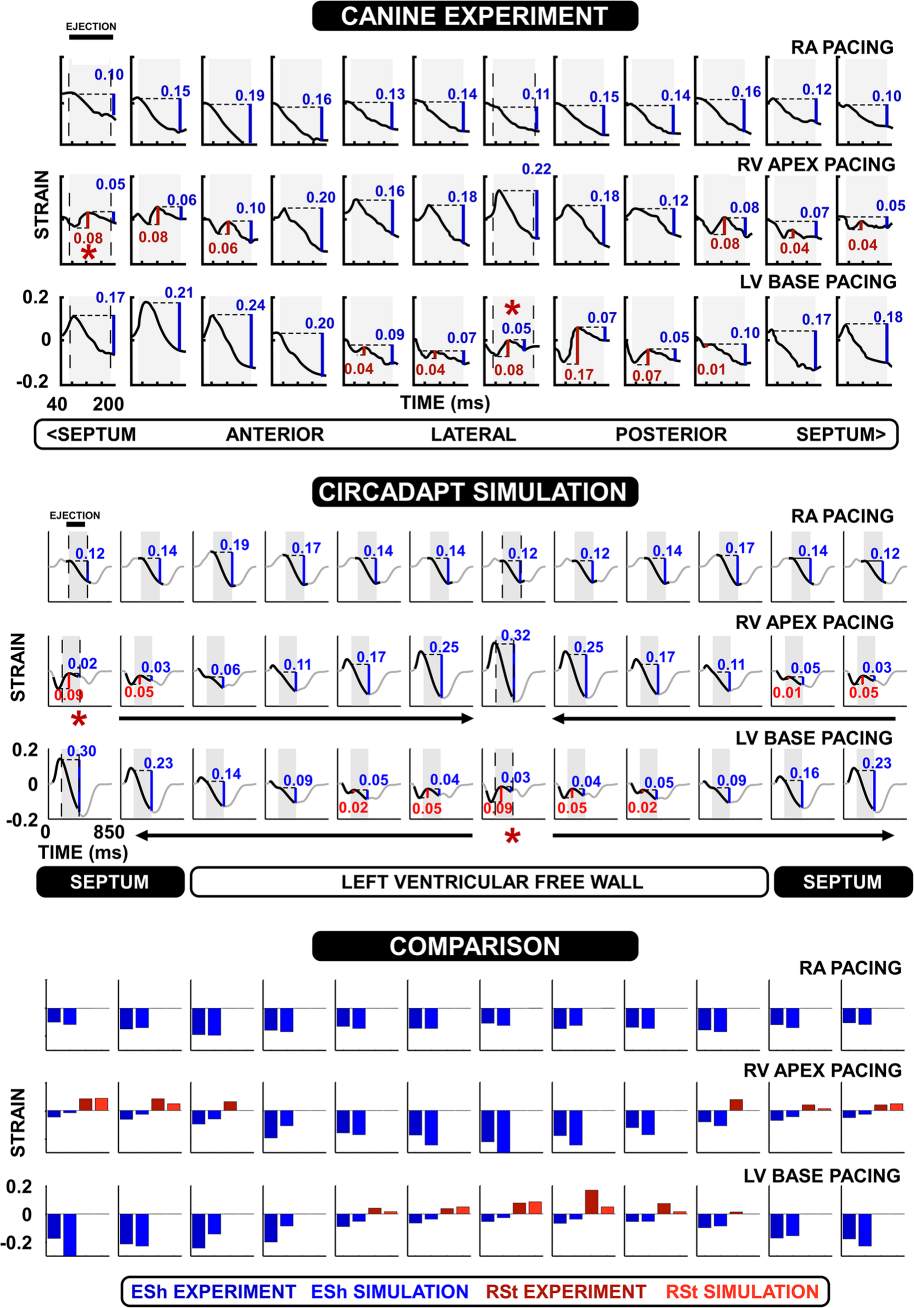
Quantitative comparison of strain patterns in canine hearts to simulations. The top panel shows experimental strain patterns during right atrial (RA), right ventricular (RV) apex, and left ventricular (LV) base pacing. The experimental strain data were originally published by Prinzen et al. (1999) [4]. The middle panel shows the corresponding CircAdapt simulations. Arrows indicate the order of activation throughout the ventricles in the RV apex and LV base simulations. Note the correspondence of the simulated patterns with the experiments, shown in black. The remainder of the simulated strain is plotted in grey. In both plots, asterisks (*) denote the approximate location of the pacing electrode, and the aortic ejection period is indicated by vertical dashed lines and a shaded area. In each panel, shortening during ejection (ESh) is shown in blue, and the rebound stretch after initial contraction (RSt) is shown in red. Experimental RSt and ESh were calculated during this study. The bottom panel plots simulated ESh (blue) and RSt (red) against values derived from the experimental data for each patch. The experimental data is on the left, and the simulated data is the right hand bar.

Strain is calculated from the CircAdapt model using the sarcomere length in each patch relative to the sarcomere length at the time of onset of ventricular activation. Simulated strain patterns during RA pacing, RV pacing and LV pacing are shown in Fig 2, together with tagged MRI strain patterns recorded from paced canine hearts by Prinzen *et al*. [4]. In both simulation and experiment, the time of activation of a segment determines its mechanical deformation pattern. The near-synchronous onset of activation in the RA pacing simulations leads to relatively uniform myocardial contraction in the LV walls. Asynchronous activation in the LV and RV pacing experiments and simulations causes early-activated regions to show pre-ejection shortening followed by stretch during systole. In the ventricular pacing simulations and the canine experiments, late-activated regions exhibit substantial degrees of pre-stretch followed by a large amount of shortening during the ejection phase. In general, early-activated regions show considerably less systolic shortening than late-activated regions.

Quantitative comparison of strain patterns between simulation and experiment is done by calculating rebound stretch (RSt) as the total amount of lengthening following initial shortening in a segment, and shortening during ejection (ESh) only, since the onset or end of shortening is not present in all experimental data. A numerical comparison between simulated and experimental strain patterns for each segment is shown in the bottom panel of Fig 2.

In RA pacing, the model predicted ESh of -0.14±0.02 (mean±standard deviation) as compared to experimental values of -0.14±0.03. We calculated the difference between the simulated and experimental ESh or RSt for each panel, and then defined ‘mean difference’ as the mean of these differences. For RA pacing the mean difference for ESh was 0.01. No RSt was present. During RV apex pacing, ESh at the earliest activated site was -0.02 and -0.05 for simulation and experiment, respectively. RSt was 0.09 in the simulation and 0.08 in the experiment. At the latest activated site, the ESh was -0.32 in the simulation and -0.22 in the experiment, with no RSt present. Across all segments the ESh was -0.13±0.1 and -0.12±0.06 for simulation and experiment, respectively, with a mean difference of 0.05. The RSt was 0.02±0.03 and 0.03±0.04 for simulation and experiment respectively, with a mean difference of 0.02. Values during LV base pacing were similar to RV apex pacing. ESh at the earliest activated site was -0.03 and -0.05 for simulation and experiment, respectively. RSt was 0.09 in the simulation and 0.08 in the experiment. At the latest activated site, the ESh was -0.3 in the simulation and -0.17 in the experiment, with no RSt present. Across all segments the ESh was -0.12±0.09 and -0.13±0.07 with a mean difference in ESh of 0.05; and RSt was 0.02±0.03 and 0.03±0.05 for simulation and experiment, respectively, with a mean difference in RSt of 0.02.

#### Asynchronous activation redistributes work from early- to late-activated regions

**Fig 3:**
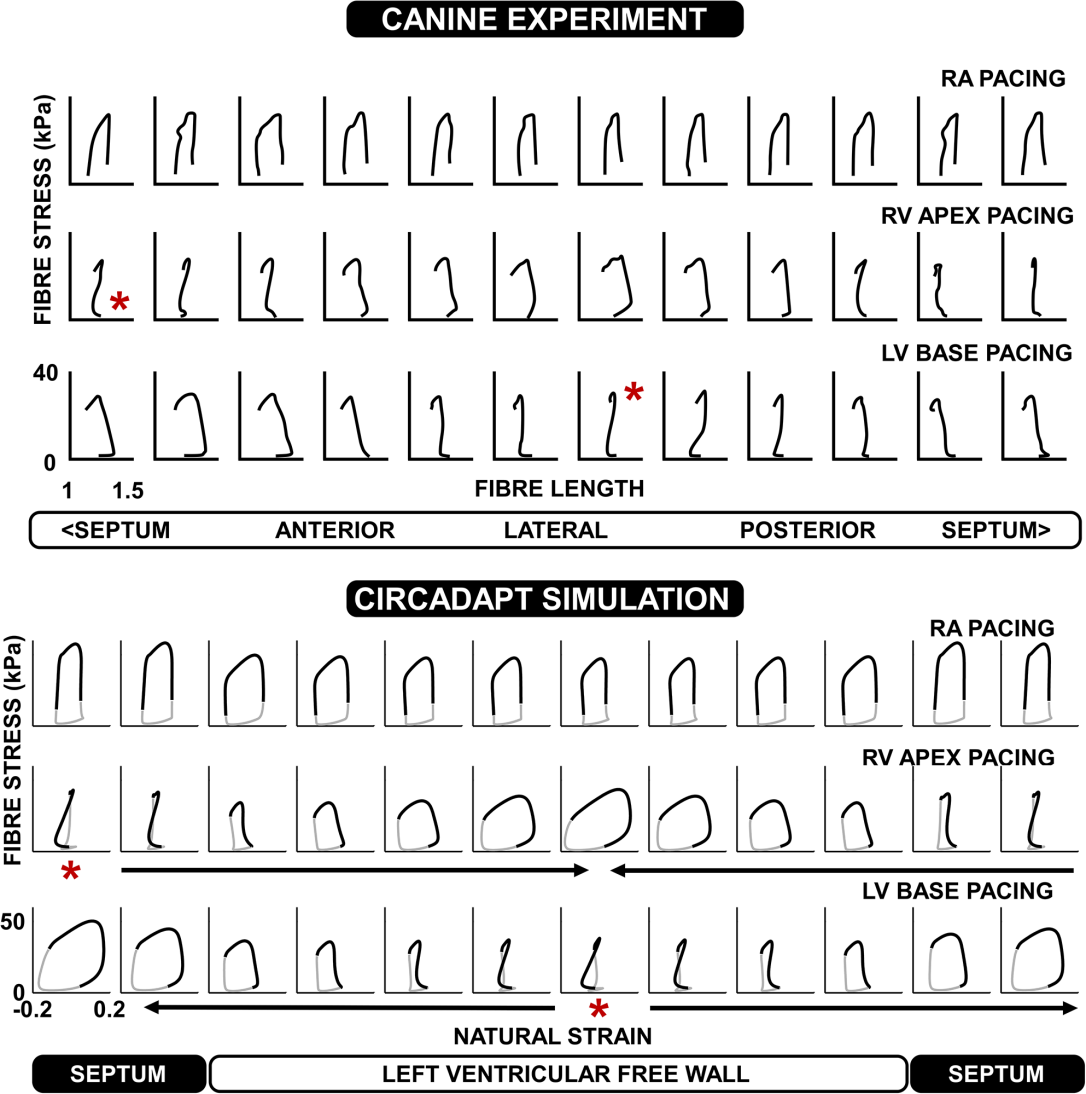
Comparison of stress-strain loops in canine hearts with simulations. The top panel shows experimental myofibre stress—length loops during RA, RV apex, and LV base pacing. The experimental data were originally published by Prinzen *et al*. (1999) [4].The bottom panel shows the corresponding CircAdapt simulations. Arrows indicate the order of activation throughout the ventricles in the RV apex and LV base simulations. Note the correspondence of the simulated patterns with the experiments, shown in black. The remainder of the simulated stress-strain loop is plotted in grey.

Simulated and experimental stress-strain plots are shown in Fig 3. During RA pacing these diagrams show a relatively uniform pattern whereas in both the ventricular pacing experiments and simulations, there is a considerable heterogeneity in the stress-strain loops throughout the ventricle. Loops with stretch during active force generation are apparent in the early-activated regions, giving a ‘figure of 8’ pattern indicative of reduced or even negative myofibre work over the whole cardiac cycle in these regions.

**Fig 4:**
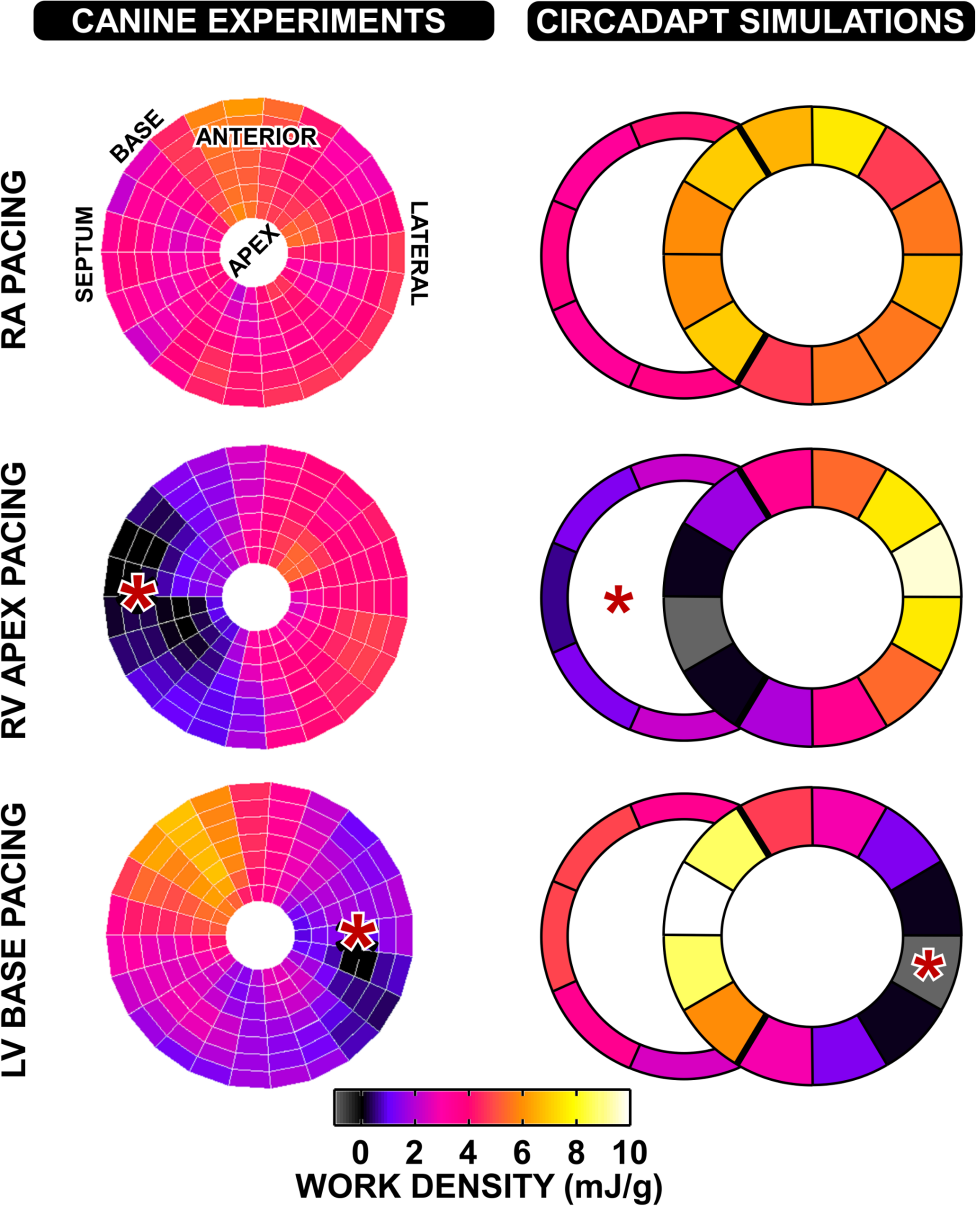
Redistribution of work in the canine heart during pacing. The left-hand panels show work density distributions within the LV during RA, RV apex, and LV base pacing (data originally published by Prinzen *et al*. 1999 [4]). Work density is plotted in mJ/g. Darker regions indicate lower work density, and lighter regions indicate higher work density. The right hand panel shows the corresponding distribution of work density from the CircAdapt simulations. The scales on the left and right hand columns are different between the two figures. The asterisks (*) denote the location of the pacing electrode.

The total work done per patch in the model is calculated as the area of the myofibre stress–natural strain loop multiplied by the volume of tissue within the patch. Regional work distributions from both simulations and experiments are shown in Fig 4, demonstrating a relatively uniform distribution of work during RA pacing. Experimental work distributions are as originally published by Prinzen *et al*. [4], using the calculation of local fibre stress described by Delhaas *et al*. [29]. The stress-strain loops from the CircAdapt model are closed since the entire cardiac cycle is simulated. During ventricular pacing, work is redistributed away from the early-activated regions towards the latest activated regions in both simulations and experiments. In the simulations, the RV performs more work during LV base pacing than RV apex pacing due to its later activation. This difference in RV work suggests a possible mechanism for the higher stroke volumes in both simulation and experiment during LV base pacing, compared to RV apex pacing.

### Patient-Specific Simulations

#### Patient-specific simulation of mechanical deformation based on endocardial activation maps

**Fig 5:**
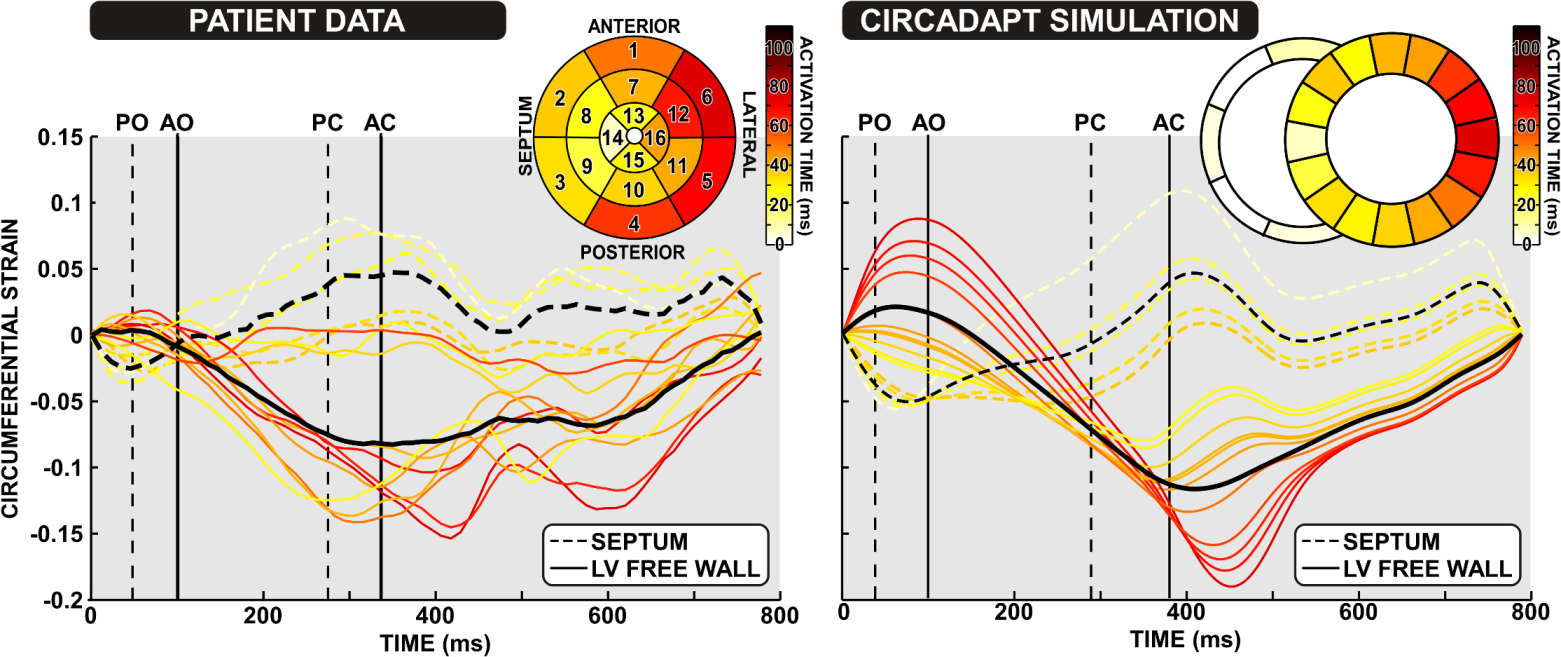
Comparison of patient tagged MRI strain with simulated deformation. Tagged-MRI strain patterns from the patient with LBBB are shown in the left hand panel, with the corresponding CircAdapt simulated strain patterns shown in the right panel. Strain patterns from the five septal segments are shown as dashed lines, and strain patterns from the LV free wall segments are shown as solid lines. Strain patterns are coloured according to their mean time of activation. The endocardial mapping activation map and the activation pattern used in the simulation are shown in the inset. The black lines denote the mean strain values from the septal and LV free wall segments. Dashed vertical lines denote pulmonary valve opening (PO) and closing (PC). Solid vertical lines denote aortic valve opening (AO) and closing (AC).

Circumferential strain patterns recorded from the patient with heart failure and LBBB using tagged MRI are shown together with the corresponding CircAdapt simulation in Fig 5. Several key features of the experimental strain patterns are visible in the simulations. Early stretch, followed by deep and synchronous shortening, is apparent in the late-activated lateral wall, whereas in the early-activated septal segments we see an initial shortening followed by stretch of the septum as the lateral wall shortens. There was, however, both more pre-stretch and dispersion in strain in the lateral wall during early systole in the simulations than in the patient recordings.

#### Comparison of patient haemodynamic measurements to simulated values

In our simulation protocol, we set the stroke volume of the patient (63 mL) as an input parameter. The simulations produced an LV end diastolic volume of 256 mL, close to the patient value of 266 mL. The LV ejection fraction in the simulations was 25%, as compared to a patient value of 24%. The simulations also produced realistic durations of opening of the aortic and pulmonary valves compared to those observed in the patient.

## Discussion

### Activation Time, Not Location, Determines Regional Fibre Strain

#### Summary

We present a lumped parameter model that enables the simulation of heterogeneity in mechanical behaviour in myocardial tissue. Our approach is based on the hypothesis that location of a region of tissue (‘patch’) within a cardiac wall determines its activation time, and that the resulting activation time then determines the myocardial deformation. Once activation time is known, the location of tissue within a wall does not have any further influence on its myocardial deformation, as the patches are connected in series through a common wall tension. We tested this hypothesis by quantitatively comparing fibre strain in simulations of paced asynchrony to circumferential strain in a paced canine model of asynchrony (Fig 2), and in a heart failure patient with LBBB (Fig 5). No fitting of parameters was performed in the pacing simulations beyond imposition of activation times.

Our simulations produced realistic deformation patterns as compared with strain measurements from tagged MRI resulting from both a paced canine model of electrical asynchrony, and from a patient with chronic heart failure and LBBB. The model successfully reproduces two important features observed in pacing and LBBB: i) early-activated regions exhibit an early shortening, followed by stretch during systole; and ii) late-activated regions exhibit pre-stretch during contraction of the early-activated regions, followed by forceful late contraction. Pre-ejection stretching of late-activated regions is caused by the shortening of early-activated regions prior to ejection. Through the Frank-Starling mechanism, pre-stretch leads to increased force of contraction, causing the systolic stretch in early regions and increased shortening in late-activated regions.

Based on the quantitative agreement between the simulation and experiment (Fig 2), we conclude that activation times, and intrinsic mechanical properties, of regions of tissue are the factors that determine myocardial deformation. The spatial location of tissue within the wall itself does not determine deformation once the tissue has been activated. Deformation of a region of tissue within a wall is affected by the mechanical load induced by other regions of tissue. This result is corroborated by the original experimental results of Prinzen *et al*. (Fig 2, top panel) showing a clear reversal of observed strain patterns in RV apex and LV base pacing.

#### Simulation of activation times

The simulated activation patterns “LV base” and “RV apex” describe the data they should be compared against, since there is no true apex or base in the model. The location of patches determines the time at which they are activated within the wall but is not used in calculations, due to our hypothesis of uniform wall tension. Thus, the simulation protocols as portrayed in Fig 1B should be interpreted carefully. This figure shows a series of patches around the ventricular cavities (see also Lumens *et al*. [16] and Huntjens *et al*. [30]). The patches are regions that make up the wall. Within each wall, the patches could be presented in any order. The visual presentation shows the motivation for the choice of timing of patch activation. For the comparison between simulated and experimental deformation, the order the simulated patches are displayed in comes from the order of activation.

The time of activation in a CircAdapt patch is the average time of activation in the myocardial tissue assigned to the patch. The total LV mid-wall activation time in the simulations of paced asynchrony was based on a total onset time of electrical activation in the pacing experiments of around 120ms [31], together with a transmural conduction time in the canine heart of 30ms [26]. Consequently, the average time of transmural activation in the earliest-activated LV patch occurs 15ms after stimulation. Similarly, the average time of activation in the latest activated patch was set 15ms before the time of latest activation recorded. The total time of LV activation used in the simulations was therefore 120 ms – 2 × 15 ms = 90 ms (Fig 1B).

#### Rationale for strain comparison

The rationale behind the method used for our comparison is that the fibre orientation in the ventricular myocardium is approximately tangent to the epicardial surface. In the middle of the myocardial wall, the fibres lie approximately circumferential to the cavity [32]. Circumferential strain from tagged MRI is calculated primarily from deformation in the middle of the wall, in the same plane as the myocytes. The strain calculated for the simulations therefore represents the circumferential strain measured in both the canine model and patients. Our comparison in the canine experiments is also based on the assumption that activation in the paced canine heart spreads uniformly away from the activation site. In the patient simulations, time of activation is known through measurements of endocardial activation time.

#### Differences between simulated and measured strain patterns

The magnitude of deformation was generally similar between the simulations and MRI recordings in both canine pacing and the patient with LBBB. However, the simulations produced a larger pre-stretch and shortening in the late-activated regions than was observed in the tagged MRI recordings in both canines and the patients. This difference may be due to the electromechanical model in CircAdapt, or it may be due to the circumferential strain in the tagged MRI recordings and fibre strain measured in the model being defined differently. Because the experimental data shown from the canine experiments comes from a mid-ventricular slice, it may not include the very earliest and latest regions, unlike the plots from the simulations. Furthermore, due to delay in the triggering of the MRI tagging, and uncertainty over when the triggering occurred relative to the ventricular activation sequence, it is also likely that our signals missed the early activation of the septum in Fig 5. This is further suggested by the apparent septal shortening at the end of the patient’s traces, although these signals will be unreliable due to deterioration of the tagging lines used for strain calculation. Hence, some early-systolic septal shortening and contralateral pre-stretch may be missing in the MRI patterns.

#### Assumption of transmurally homogeneous fibre strain

The original CircAdapt model was based on the assumption that fibre stress and fibre strain within the myocardial walls of the heart are approximately homogeneous [24,33]. This is based on experiments showing no significant transmural differences in strain along the myofibre direction [34], suggesting that in the healthy heart mechanical loading is homogenised throughout the ventricular myocardium *via* remodelling of myofibre alignment [35]. In the MultiPatch module, this assumption is applied to the patches, so that (Cauchy) fibre stress and fibre strain are homogeneous within a patch, but can vary between patches in a wall. Patches within a wall are mechanically coupled through wall tension and conservation of energy. The assumption of homogeneity of transmural fibre strain has been challenged by experimental results from Ashikaga *et al*. in canine hearts under both sinus rhythm and epicardial pacing [36,37], and by finite element simulations showing transmural dispersion in time to onset of shortening [38,39]. In general, clinical and experimental measurements investigating transmural variation in strain patterns is difficult to obtain, and most studies in the literature represent averaged myofibre behaviour, as represented in the MultiPatch model.

#### Redistribution of work

We also compared stress-strain loop morphology and work distribution in the paced canine simulations and experiments (Figs 3 and 4). By calculating segmental work in the CircAdapt model, we observe a redistribution of work in pacing-induced mechanical dyssynchrony. Work is redistributed away from early-activated regions towards late-activated regions as previously reported [4,40]. The smaller decrease in stroke volume arising from LV base pacing than RV apex pacing can be explained by an increased contribution of work performed by the RV, as previously described by Lumens *et al*. [16], and consistent with the experimental findings of Quinn *et al*. in a porcine model [41]. The absolute values of haemodynamic parameters in the simulations are different to the canine experiments since the standard CircAdapt model represents human physiology, but the relative changes between RA pacing and ventricular pacing are similar.

### MultiPatch Allows Fast Simulation of Asynchronous Activation

Simulation of cardiovascular mechanics using the MultiPatch module requires a considerably reduced computational effort compared to FE implementations. Speed of the Matlab implementation can be assessed using CircAdapt files provided as an online supplement that perform all simulations in the present study (.txt files). FE-based models of cardiac mechanics presently run significantly slower than real-time and generally require access to supercomputing facilities. The speed of simulation in CircAdapt allows assessment of the role of factors that require simulation of multiple full cardiac cycles, such as homeostatic control and tissue adaptation [24,28]. Real time simulation on regular desktop computers when implemented in C++ enables use of CircAdapt in a teaching environment [42]. A future application of the MultiPatch module is integration within the CircAdapt Simulator (freely available through www.circadapt.org) to allow education of clinical trainees in the haemodynamic consequences of myocardial scar and conduction disorders, and in the interpretation of regional deformation patterns in the failing heart.

Our study focusses on the heart. However, the theory and methods could also be applied to computer models of other contractile organs such as the gastro-intestinal tract or uterus to allow faster simulation [43,44].

### Patient-Specific Simulation of Ventricular Asynchrony

In the current study, we have established that the MultiPatch module in CircAdapt enables simulation of realistic deformation patterns in a patient-specific manner, based on a series of relatively simple parameter changes in the model (Fig 5) and invasively recorded activation maps. Combined with the speed at which simulations can be performed in CircAdapt, this provides the opportunity for patient-specific simulations of mechanical dyssynchrony and haemodynamics that can be performed on a personal computer in a bedside context. We have focussed on the effects of asynchronous mechanical activation on deformation in this study, but the model is also capable of simulating regional wall hypocontractility and/or stiffening, consistent with acute or chronic regional myocardial infarction or fibrosis [30]. By simulating CRT combined with patient-specific myocardial properties obtained by fitting to observed myofibre strain, it may be possible to predict patient response to CRT.

### Limitations

The ventricular geometry within the CircAdapt model is highly simplified. In particular, we assume that the curvature is the same at each point in a wall, which is not the case in the real heart. Mechanical effects of the atrioventricular rings on ventricular tissue are not included. Geometric simplifications in the CircAdapt model mean that circumferential, longitudinal, and fibre strain are considered to be equivalent, and so no apex-base axis is present. Our simplified approach prevents inclusion of transmural differences in electromechanical properties [45], and simulation of mechanical shear.

### Conclusions

The MultiPatch module integrated within the CircAdapt model of the heart and circulation produces realistic simulations of local mechanical deformation, work distribution, and global haemodynamic pump function of the dyssynchronous heart. The success of the model in predicting fibre strain patterns suggests that once timing of activation of a region of tissue is known, the location of the tissue is not important for determining of myocardial deformation. We have demonstrated the potential of this model for use in patient-specific simulation of dyssynchronous heart failure, based on clinical recordings of the LV endocardial activation sequence. As a future perspective, comparing patient-specific simulations from the MultiPatch module against recorded myocardial deformation could be used to determine whether the tissue substrate underlying electro-mechanical dyssynchrony in a patient is amenable to CRT.

## Materials and Methods

### Ethics Statement

The patient included in the simulation part of the study was part of the CARTO-CRT trial and provided a written informed consent for the study. The CARTO-CRT trial protocol was approved by the CHU-Bordeaux ethics committee (registered at clinicaltrial.gov: NCT01270646).

### Endocardial Electro-Anatomical Mapping

A deflectable-tip catheter was inserted into the left ventricle through a trans-septal route (Navistar catheter, Biosense Webster, Diamond Bar, CA). Left ventricular endocardial electro-anatomical mapping was performed during sinus rhythm using Carto V3 (Biosense Webster, Diamond Bar, CA). After the reconstruction of the left ventricular geometry, detailed activation mapping was conducted with a minimum of 100 equally distributed points. All points were reviewed manually to ensure the quality of the activation map. The location of the LV septum, the anterior, lateral and posterior wall, the mitral annulus and the apex were defined on the CARTO anatomical mesh. The AHA segmentation was then used to accurately localise each of the endocardial activation points. Mean activation times were then applied to the AHA segmentation as described in the online supplement, section ‘Aligning CARTO data with an AHA segmentation of the left ventricle’.

### Tagged MRI Strain Measurements

The MRI study was conducted on a 1.5 Tesla clinical device (Magnetom Avanto, Siemens Medical Systems, Erlangen, Germany) equipped with a 32-channel cardiac coil. Myocardial tissue tagging was performed using complementary spatial modulation of magnetization (CSPAMM) combined with steady state free precession cine imaging [46]. Images were acquired in three parallel short-axis planes at the basal, mid, and apical levels of the left ventricle. A multiple expiratory breath hold scheme was used to enable strain imaging at high temporal resolution (11 ms). Sequence parameters were: prospective triggering, repetition time 4.7 ms, echo time 2.3 ms, bandwidth = 369 Hz/pixel, flip angle = 20°, FoV 300 × 300 mm, matrix size 256 × 78, slice thickness 6 mm, tag spacing 7 mm. CSPAMM images were processed using the software Osirix v3.9.4 (Osirix Fondation, Geneva, Switzerland). Strain computation was performed with the Sine Wave Modeling method [47] using the inTag Osirix plugin (CREATIS-INSA, Lyon, France). Circumferential strain curves were computed for each of the 16 left ventricular segments, according to the standard AHA segmentation, excluding the apical segment [27].

### Calculating Wall Tension and Transmural Pressure in the TriSeg Module

**Fig 6:**
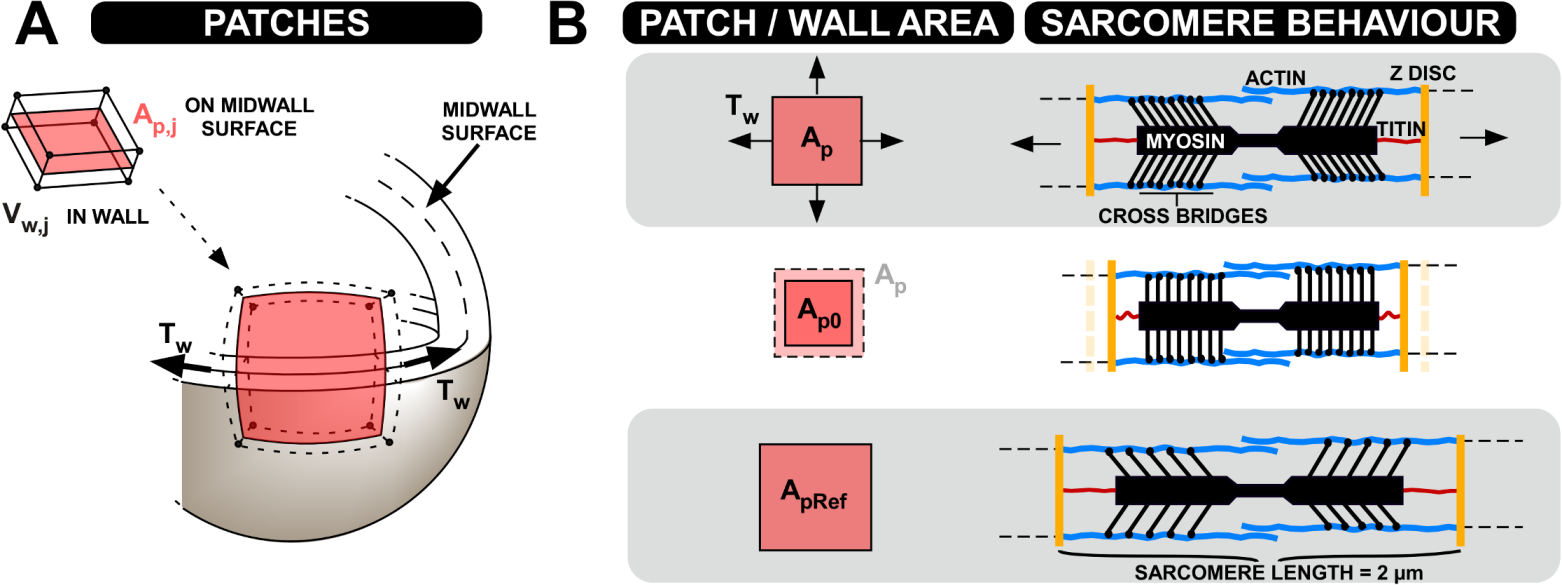
Concepts behind the MultiPatch model. Panel A represents one of the patches making up a wall, with its area A_p,j_ being defined on the mid-wall surface. The mid-wall tension T_w_ acts on this surface. The patch is also assigned a volume V_p,j_. Panel B demonstrates the difference between the patch areas A_p,j_, A_p0,j_, and A_pRef,j_ in terms of sarcomere behaviour.

A summary of the notation used in this section is provided in Table 2. Before describing the MultiPatch model, we recapitulate how wall tension is calculated in a uniform wall, as originally described for the TriSeg model [25], simulating three ventricular walls, encapsulating two cavities. The volume of blood in the cavity *V*_*c*_ is used to derive the area *A*_*w*_ of the mid-wall surface on which wall tension is calculated as described by Lumens *et al*. [25]. The mid-wall surface is the surface dividing the wall volume in half in the radial direction, as shown in Fig 6. Natural fibre strain *ε*_*f*_ is calculated from *A*_*w*_ assuming zero-strain reference for that area to be *A*_*w*,*ref*_, the wall area when sarcomere length is 2μm:

$$\varepsilon_{f}=\frac{1}{2}\text{ln}\left(\frac{A_{w}}{A_{w,\text{Ref}}}\right)$$ (1)

**Table 2:** Summary of notation used.

**Variable name**	**Units**	**Meaning**
T	Nm^-1^	wall tension
p_trans_	Pa	transmural pressure
V	cm^3^	volume
C_m_	cm^-1^	mid-wall curvature
A	cm^2^	surface area
*ε*_*f*_	-	natural fibre strain
*E*	-	engineering fibre strain
*σ*_*f*_	Pa	fibre stress
**Subscript name**	**Meaning**	
*m*	mid-wall surface	
*w*	wall	
*p*	patch	
*i*	intrinsic sarcomere length	
*j*	index of patch	
*Ref*	reference at sarcomere length 2μm	

Fibre stress *σ*_*f*_ was calculated as a function of strain *ε*_*f*_, using a phenomenological model based on physiological experiments, as explained in the sarcomere module originally published by Lumens *et al*. [25] (online supplement, section ‘The CircAdapt sarcomere contraction model’). To calculate the wall tension *T*_*w*_, considered to be concentrated on the mid-wall surface, Lumens *et al*. used the following relation derived from the principle of conservation of energy (online supplement, section ‘Conservation of energy’):

$$T_{w}=\frac{\sigma_{f}(\varepsilon_{f})V_{w}}{2A_{w}}\;.$$ (2)

The symbol *V*_*w*_ refers to the wall volume. In the TriSeg model, the geometries of the three ventricular walls are iteratively calculated to satisfy mechanical equilibrium between the walls on their common intersection curve. As a result, wall areas *A*_*w*_ and wall curvatures *C*_*m*_ can change within the TriSeg iteration. Once the wall tension and curvature are known, the transmural pressure can be calculated using Laplace’s law,

$$P_{trans}=2T_{w}C_{m},$$ (3)

where *C*_*m*_ is the mid-wall curvature, calculated from the geometry of either the chamber or the TriSeg module as described by Lumens *et al*. [25]. The wall tension in Eq 2 is the same as that in Laplace’s law (Eq 3), with units N/m, and is distinct from tensile force (units N).

### Calculating Wall Tension Using the MultiPatch Module

In the MultiPatch module, a wall is subdivided into *n* patches, indexed *j*. Each patch is assigned properties including a reference area *A*_*pRef*,*j*_ (Fig 6) and tissue volume *V*_*p*,*j*_. Tissue properties, and hence fibre Cauchy stress and strain, are allowed to vary between patches, and so Eq 2 no longer holds throughout the wall. To solve the related equilibrium equations, wall tension *T*_*w*_ is linearized about a working point corresponding to the wall area at zero wall tension (*A*_*0*,*w*_), *i*.*e*. wall area if the tissue would experience no external load;

$$T_{w}(A_{w})\approx \frac{dT_{w}}{dA_{w}}\left(A_{w}-A_{0,w}\right)\;.$$ (4)

*A* *_0,w_* and wall area stiffness d*T*_*w*_/d*A*_*w*_ are calculated using the MultiPatch module so that Eq 4 can be used to calculate *T*_*w*_.

For each patch we use a linearized relation for the wall tension in one patch *T*_*p*,*j*_ as a function of patch mid-wall area *A*_*p*,*j*_ (Fig 6), equivalent to that for the wall in Eq 4:

$$T_{p,j}(A_{p,j})\approx \frac{dT_{p,j}}{dA_{p,j}}\left(A_{p,j}-A_{p0,j}\right)$$ (5)

The mid-wall surface of the patch is assumed to lie on a spherical surface, as in the TriSeg model. Since transmural pressure and curvature are common to all patches in a wall, by application of Laplace’s law it follows that the wall tension *T*_*p*,*j*_ must be the same for all patches in the wall, and equals *T*_*w*_. If wall tension equals zero, the mid-wall area of the whole wall equals the sum of zero wall tension mid-wall areas of all patches, resulting in a value for *A*_*0*,*w*_, to be substituted into Eq (4) for the whole wall:

$$A_{0,w}=\sum_{j=1}^{n}A_{p0,j}$$ (6)

We can differentiate Eq 6 with respect to wall tension. Taking the inverse of the resulting expression, we find that the total wall stiffness d*T*_*w*_/d*A*_*w*_ in Eq (3) equals the inverse of the sum of inverse stiffness of all patches:

$$\frac{dT_{w}}{dA_{w}}=1/{\sum_{j=1}^{n}\left(\frac{dT_{p,j}}{dA_{p,j}}\right)}^{-1}$$ (7)

*A*_*0*,*w*_ and d*T*_*w*_/d*A*_*w*_ as calculated in Eqs 6 and 7 are then used in Eq 4 to determine *T*_*w*_ for the current value of *A*_*w*_ in the TriSeg iterative scheme. *A*_*0*,*w*_ and d*T*_*w*_/d*A*_*w*_ do not change between TriSeg iterations and so need only be calculated once per time step. Once the TriSeg solution is found, transmural pressure *p*_*trans*_ can be calculated by Laplace’s law (Eq 4) using *T*_*w*_ and *C*_*m*_. In this way, mechanical behaviour of the total wall is described in relation to the properties of its composing patches.

After wall tension has been determined, the true areas *A*_*p*,*j*_ of the separate patches are calculated using *T*_*w*_, d*T*_*p*,*j*_/d*A*_*p*,*j*_, and *A*_*p0*,*j*_ in Eq (5). The fibre stress *σ*_*f*,*j*_ and fibre natural strain *ε*_*f*,*j*_ are then calculated for each patch, using the patch equivalent of Eq (1) and the sarcomere module (online supplement, section ‘The CircAdapt sarcomere contraction model’). Different mechanical properties and activation times may be assigned to each patch and so can result in differences in fibre stress and strain between patches, despite having the same wall tension and curvature. The thickness of a patch can be calculated by dividing its constant volume *V*_*p*,*j*_ by its time-dependent area *A*_*p*,*j*_.

### Calculating Patch Stiffness and Unloaded Area

We now explain how *A*_*p0*,*j*_ and *dT*_*p*,*j*_ */ dAp*,*j* are calculated for use in Eqs 6 and 7. In the CircAdapt sarcomere module, sarcomere length *L*_*s*_ is described as follows,

$$L_{s,j}=L_{si,j}+L_{se,j},$$ (8)

where *L*_*se*,*j*_ is the length of the series elastic element and *L*_*si*,*j*_ is the intrinsic sarcomere length (online supplement, section ‘The CircAdapt sarcomere contraction model’). *L*_*se*,*j*_ is proportional to the active stress generated by the tissue. Assuming that area is proportional to the square of sarcomere length, *L*_*s*,*j*_ is related to patch area *A*_*p*,*j*_ by

$$A_{p,j}={\left(\frac{L_{s,j}}{L_{\text{sRef,j}}}\right)}^{2}A_{\text{pRef,j}}\;.$$ (9)

*A*_*pRef*,*j*_ is the reference area of patch *j* when the sarcomere length is *L*_*sRef*,*j*_ (*i*.*e*. 2μm, see Fig 6).

In order to calculate *A*_*p0*,*j*_, we use Eq 5. Since *A*_*w*_, and hence *A*_*p*,*j*_, can change during the TriSeg iteration, *L*_*s*_ is unknown. We therefore use the intrinsic sarcomere length *L*_*si*_ with Eq 9 to compute a first estimate of the patch area, *A*_*pi*,*j*_. *A*_*pi*,*j*_ corresponds to the area of the patch without the influence of active stress. The first estimate of natural fibre strain in the patch is

$$\varepsilon_{fi,j}=\text{ln}\left(\frac{L_{si,j}}{L_{\text{sRef,j}}}\right)\;.$$ (10)

The strain *ε*_*fi*,*j*_ is used by the CircAdapt sarcomere module to calculate the fibre stress *σ*_*fi*,*j*_ and fibre stiffness *dσ*_*fi*,*j*_ */ dε*_*fi*_ arising from *L*_*si*,*j*_. *σ*_*fi*,*j*_ is a component of the fibre passive stress. The stiffness *dσ*_*fi*,*j*_ */ dε*_*fi*_ additionally depends on the contractile state of the tissue in the patch since the contractile and passive elements of the sarcomere module are arranged in parallel (online supplement, Eq. 21). Hence *dσ*_*fi*,*j*_ */ dε*_*fi*_ is dependent on the timing of patch activation.

To calculate the unloaded area *A*_*p0*_, we must subtract the effects of passive stress due to *L*_*si*_ from *A*_*pi*,*j*_. By the principle of conservation of energy (Eq 2), *σ*_*fi*,*j*_ and area *A*_*pi*,*j*_ must induce a part *T*_*pi*,*j*_ of the wall tension *T*_*w*_ felt by the patch,

$$T_{pi,j}=\frac{\sigma_{fi,j}V_{p,j}}{2A_{pi,j}}\;,$$ (11)

where *V*_*p*,*j*_ is the wall volume assigned to patch *j*.

The patch area stiffness d*T*_*pi*,*j*_ /d*A*_*pi*,*j*_ is found by the chain rule:

$$\frac{dT_{pi,j}}{dA_{pi,j}}=\frac{dT_{pi,j}}{d\varepsilon_{fi,j}}/\frac{dA_{pi,j}}{d\varepsilon_{fi,j}}\;.$$ (12)

Substitution of Eqs (9,10 and 11) into Eq (12), and taking the derivative gives:

$$\frac{dT_{pi,j}}{dA_{pi,j}}=\left(\frac{d\sigma_{fi,j}}{d\varepsilon_{fi,j}}-2\sigma_{fi,j}\right)\frac{V_{p,j}}{4A_{pi,j}{}^{2}}\;.$$ (13)

We can now calculate the mechanically unloaded patch area *A*_*p0*,*j*_ using Eq 5 by

$$A_{p0,j}=A_{pi,j}-T_{pi,j}/\frac{dT_{pi,j}}{dA_{pi,j}}\;.$$ (14)

**Fig 7:**
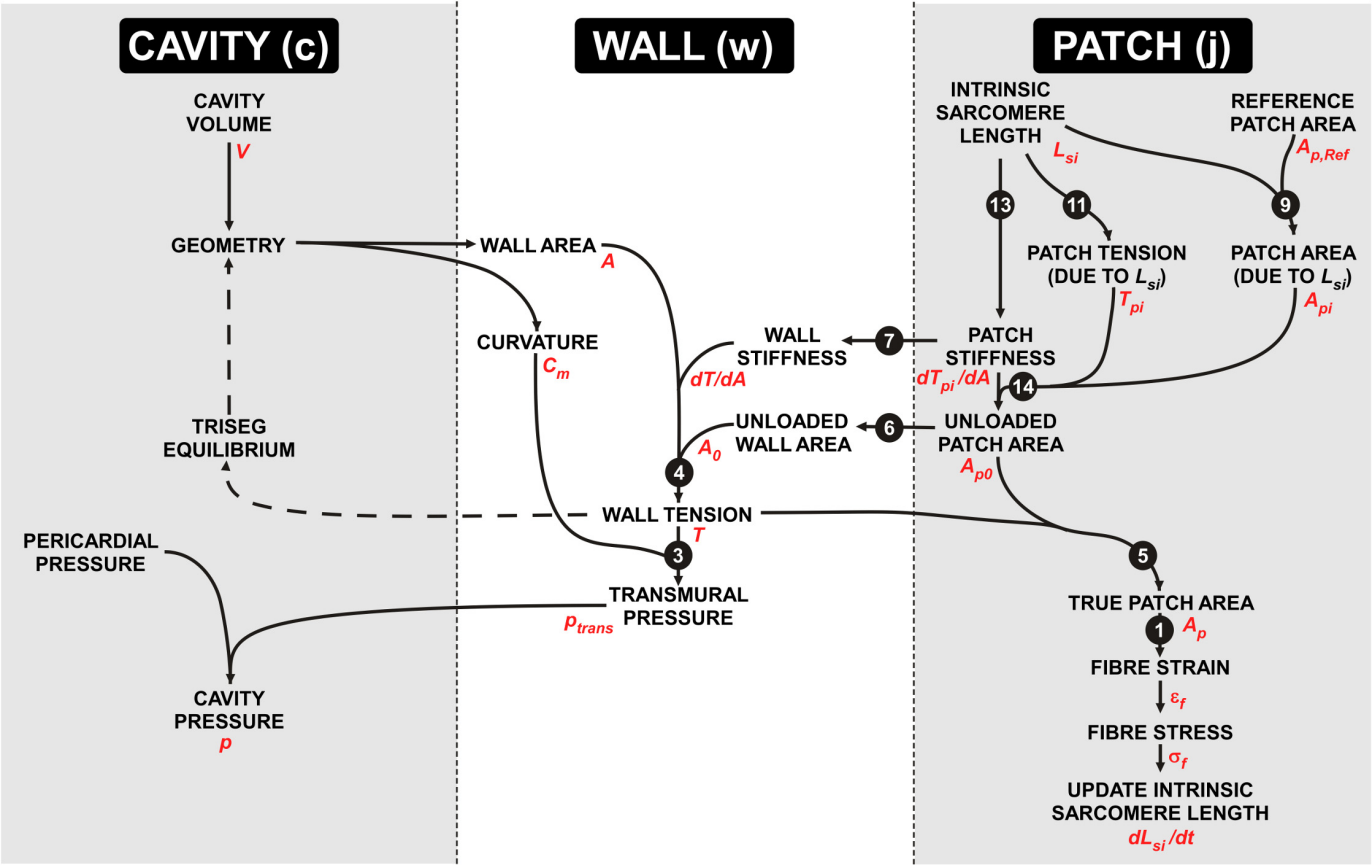
The sequence of calculations using the MultiPatch model. Calculations are divided into three levels–cavity, wall, and patch. Calculated quantities are given in black text. The names of the variables used in the text are shown in red. Black arrows show the sequence in which calculations are performed. White numbers indicate the equation number in the text used for calculation.

A schematic showing calculations performed in the cavities, patches, and walls is given in Fig 7.

### Baseline Simulation of the Human Heart and Circulation

The CircAdapt model was used to obtain a simulation that represents the healthy human cardiovascular system. A resting cardiac output of 5.1 l/min and heart rate of 70bpm, and an exercise condition of three times cardiac output and doubled heart rate, were used to adapt tissue volumes and areas in the cardiac walls and large blood vessels as described previously [24,28]. Mean arterial pressure of 92 mmHg was maintained at both rest and exercise. The resulting baseline human simulation was used as the basis for all subsequent simulations. All parameters used in the reference simulation may be found in the PRef Matlab structure that is part of the code provided in the online supplement (.txt files).

### Comparing Simulated and Experimental / Clinical Data

Strain for comparison to MRI data is calculated by taking the model sarcomere length at a reference time point, *L*_*s*_ *(t*_*0*_ *)*, for each patch, then computing the strain in each patch *E*_*j*_ as

$$E_{j}=\frac{L_{s,j}}{L_{s,j}\left(t_{0}\right)}-1$$ (15)

*E_j_* is non-dimensional. For the canine simulations, the reference time is the time of first ventricular activation. For the patient simulation, the reference time is 100ms before aortic valve opening.

## Supporting Information

S1 Text MultiPatch Paper Supplement. This document contains a description of placing mean activation times on the standard AHA segmentation, a description of the sarcomere model in CircAdapt, and an illustration of the calculation loop in CircAdapt.
(DOCX)

S2 Text README. Contains instructions for unpacking the CircAdapt code and running the simulations in the paper.
(TXT)

S3 Text Unpack CircAdapt. Unpacks the CircAdapt model and simulation software (see S2 Text).
(TXT)

S4 Text Patient Data Builder. Builds the patient strain data structure used when generating the figures (see S2 Text).
(TXT)

S5 Text PRef Builder. Builds the PRef structure used in the simulations (see S2 Text).
(TXT)

S6 Text CircAdapt for Release. The version of the CircAdapt model used for these simulations (see S2 Text).
(TXT)

S7 Text CircAdapt Tools. Tools for use with CircAdapt (see S2 Text).
(TXT)

S8 Text MultiPatch Paper Simulations. Code for simulations in the paper and figure generation (see S2 Text).
(TXT)

S9 Text CircAdaptP Manual. Manual for using the enclosed CircAdapt model.
(DOC)
