# Supplementary material for: Fast Simulation of Mechanical Heterogeneity in the Electrically Asynchronous Heart Using the MultiPatch Module
Source: PLoS Comput Biol. 2015 Jul 23;11(7):e1004284. doi: 10.1371/journal.pcbi.1004284 (PMC4512705; doi:10.1371/journal.pcbi.1004284)
Supplement: S1 Text — This document contains a description of placing mean activation times on the standard AHA segmentation, a description of the sarcomere model in CircAdapt, and an illustration of the calculation loop in CircAdapt. (DOCX) [file pcbi.1004284.s001.docx]

Fast simulation of mechanical heterogeneity in the electrically asynchronous heart using the MultiPatch module

**Online data supplement**

# Aligning CARTO data with an AHA segmentation of the left ventricle

Points lying on the septum were identified in the CARTO system by an experienced clinical operator. The apex – base axis was identified using principal component analysis through all points. The cartesian co-ordinates of each point were then converted to polar co-ordinates using the apex-base axis as the z-axis. The angle theta = 0 was taken to be at the location of the centre of mass of the identified septal points. The resulting polar co-ordinate system was then used to identify the AHA segments by dividing the CARTO points into an apical, mid and basal region. The apical region was from 12.5% to 37.5% of the total range of the points along the z-axis, the mid region was from 37.5% to 62.5% and the basal region was from 62.5% to 87.5%. The lowest ( 0% -12.5%) and uppermost (87.5% - 100%) of the total range was excluded from the analysis.

The apical region was sub-divided into four segments as in the standard AHA segmentation, each of 90. 0 was taken to be the centre of the septal segment. The mid and basal regions were each divided into six segments of 60. Again, 0 was taken to be at the centre of the septal segment. Activation time in the resulting 16 segment division of the left ventricle was then calculated for each segment by taking the mean of the activation time over all triangles within the CARTO mesh lying within each segment.

# The CircAdapt sarcomere contraction model

The contraction model currently used in CircAdapt is a modified Hill model based upon the one presented by Lumens *et al[1]*. The model aims to reproduce basic properties of length dependent activation in cardiac tissue[2,3]. The fibre stress is determined by the rise of contractility in the fibre (representing density of cross bridge formation) and the fibre strain. The fibre model is divided into an active and passive stress component, with the active stress arising from myofibre contraction, and the passive stress component arising from the soft tissue deformation of the myocardium.

The current myofibre strain is used to compute the sarcomere length in the model. In CircAdapt, natural myofibre strain *ε_f_* in a patch is defined as

|  | $\varepsilon_{f}= \ln\frac{L_{s}}{L_{s,R\text{ef}}}$ , | (1) |
| --- | --- | --- |

where *L_s_* is the total sarcomere length, and *L_s,Ref_* is the reference sarcomere length of 2µm. From the strain we can therefore calculate the sarcomere length as

|  | $L_{s}= L_{s,\text{ref}} \text{exp}\left( \varepsilon_{f} \right)$ | (2) |
| --- | --- | --- |

#### Fibre active stress

The fibre active stress is determined by a modified Hill model controlled by two variables, the intrinsic sarcomere length *L_si_* and the contractility *C*. The governing equation for *L_si_* is

|  | $\frac{dL_{si}}{dt}= v_{\text{max}} \left( \frac{L_{s}- L_{si}}{L_{se,\text{iso}}}-1 \right)$, | (3) |
| --- | --- | --- |

where *L_s_ – L_si_* is the length of the series elastic element in the Hill model, and *L_se,iso_* is the length of the series elastic element during isovolumetric contraction. The length of the series elastic element represents the deformation of the sarcomere due to stretch of cross bridges under mechanical load during contraction.

Contractility is a phenomenological parameter representing the density of cross bridge formation within the fibres in the current patch. The contractility is determined by the following differential equation,

|  | $\frac{dC}{dt}= \frac{1}{\tau_{\text{rise}}} C_{L}\left( L_{si} \right) F_{\text{rise}}\left( t \right)- \frac{1}{\tau_{\text{decay}}} C \text{g(X}$), | (4) |
| --- | --- | --- |

where,

|  | $\tau_{\text{rise}}=0.55 T_{r}t_{A},$ | (5) |
| --- | --- | --- |
|  | $\tau_{\text{decay}}=0.33 T_{d}t_{A}$. | (6) |

*T_r_* and *T_d_* are constants, and *t_A_* is the duration of activation of the fibre. *t_A_* depends on the sarcomere extension,

|  | $t_{A}=0.65+1.057 \frac{L_{si}}{L_{si,0}}$. | (7) |
| --- | --- | --- |

*C_L_* describes the increase in cross bridge formation with intrinsic sarcomere length due to an increase in available binding sites,

|  | $C_{L}\left( L_{si} \right)=\tanh\left( 4 \left( L_{si}- L_{si,0} \right)^{2} \right)$. | (8) |
| --- | --- | --- |

*F_rise_(t)* is a phenomenological representation of the rate of cross bridge formation within the patch,

|  | $F_{\text{rise}}\left( t \right)=0.02 x^{3}\left( 8-x \right)^{2}\text{exp}\left( -x \right)$, | (9) |
| --- | --- | --- |

where

|  | $x\left( t \right)=\text{min}\left( 8, \text{max} \left( 0, \frac{t_{\text{c}}}{\tau_{\text{rise}}} \right) \right)$, | (10) |
| --- | --- | --- |

and *t_c_ = t - t_act_*, where *t_act_* is the time of onset of activation of the patch, *i.e.* the time at which the first myocytes within the patch begin to form cross bridges in response to electrical activation.

The decay term in equation (4) gives an exponential decay in the contractility. This decay is delayed by the term *g(X)*. The term *g(X)* is an approximation of the function *tanh(X)* using a sine curve to ensure that it takes value 0 or 1 outside of the region where it exhibits a large change,

|  | $\text{g(X)}=0.5+0.5 \sin\left( \text{sign}\left( X \right) \text{min}\left( \frac{\pi}{2}, \text{abs}\left( X \right) \right) \right)$, | (11) |
| --- | --- | --- |

where,

|  | $X=\frac{t_{c}- t_{A}}{\tau_{d}}.$ | (12) |
| --- | --- | --- |

The effect of the formulation for contractility is as follows: as the chamber wall is stretched by an expanding volume of blood, the series elastic element (*L_se_*) lengthens, causing a corresponding lengthening of the contractile element (*L_si_*). Given an onset of cross bridge formation in response to electrical excitation of parts of the patch at time *t_act_*, the contractility *C* begins to rise according to *F_rise_(t)*. The longer the contractile element *L_si_*, the greater both the duration of the contractile phase (equation (7)) and the rate of  increase in contractility (equation (8)) are. Once the duration of activation, *t_act_*, is over, the contractility begins to decay exponentially.

We use the following equations to convert contractility and sarcomere length into actively generated fibre stress *σ_f,actT_* within a patch,

|  | $\sigma_{f,\text{actT}}=\sigma_{f,\text{act}}C(L_{si}-L_{si,\text{ref}})\frac{L_{se}}{L_{se,\text{iso}}}$ , | (13) |
| --- | --- | --- |

where *σ_f,act_* is a parameter and *L_se_* / *L_se,iso_* is the extension of the series elastic element. Hence the actively generated fibre stress is determined by the stretching of the myosin heads in response to sarcomere shortening multiplied by the number of cross bridges formed, which is the contractility multiplied by the sarcomere extension from reference ( *C ( L_si_ - L_si,ref_)* ).

#### Fibre passive stress

Passive deformation of the soft tissue making up the myocardium will also generate stress within the walls, *σ_f,pasT_*. In CircAdapt, this is considered to be a passive stress in the fibres in each patch. This contains two components, the stress arising from the myocytes themselves due to internal structures such as titin anchoring to the Z disc (*σ_f,tit_*), and the stress arising from the extracellular matrix surrounding the myocytes (*σ_f,ECM_*). Hence,

|  | $\sigma_{f,pasT}=\sigma_{f,tit}+ \sigma_{f,ECM}.$ | (14) |
| --- | --- | --- |

The extension of the cells for the passive stress calculation *λ_s,pas_* is done relative to a different reference length *L_s0,pas_* as follows,

|  | $L_{s,pas}=\frac{L_{s0}}{L_{s0,pas}}\text{exp}\left( e_{f} \right)$. | (15) |
| --- | --- | --- |

The ECM is modelled as being stiffer than the contribution due to cellular structures such as titin,

|  | $\sigma_{f,ECM}=0.0349 \sigma_{f,pas} \left( {\lambda_{s,pas}}^{10}-1 \right)$, | (16) |
| --- | --- | --- |

where *σ_f,pas_* is a parameter.

The passive stress in the patch due to cellular structures such as titin is modelled as being softer than the ECM, and is governed by the following equation

|  | $\sigma_{f,tit}=0.01 \sigma_{f,act} \left( {\lambda_{s,pas}}^{k}-1 \right)$ | (17) |
| --- | --- | --- |

Where the parameter *k* is given by

|  | $k=\frac{2L_{s,ref}}{{dL}_{s0,pas}}$, | (18) |
| --- | --- | --- |

and *dL_s0,pas_* is a parameter. Using equations (13) and (14) we then arrive at the following expression for fibre stress within a patch,

|  | $\sigma_{f}= \sigma_{f,actT}+ \sigma_{f,pasT}$. | (19) |
| --- | --- | --- |

#### Derivative of fibre stress with respect to fibre strain

As described in the main article, calculating the compliance in a patch requires the stiffness $\frac{d\sigma_{f}}{de_{f}}.$ We see from equations (15), (16) and (17) that,

|  | $\frac{d\sigma_{f,pasT}}{de_{f}}=$ $0.349 \sigma_{f,pas} {\lambda_{s,pas}}^{10}+0.01 k \sigma_{f,act}{\lambda_{s,pas}}^{k}$. | (20) |
| --- | --- | --- |

and we can calculate $\frac{d\sigma_{f,actT}}{de_{f}}$ using equation (13), equation (2), and the relation *L_se_ = L_s_ – L_si_*,

|  | $\frac{d\sigma_{f,actT}}{de_{f}}= \sigma_{f,\text{act}}C(L_{si}-L_{si,\text{ref}})\frac{L_{s}}{L_{se,\text{iso}}}$. | (21) |
| --- | --- | --- |

We then have,

|  | $\frac{d\sigma_{f}}{de_{f}}=\frac{d\sigma_{f,actT}}{de_{f}}+ \frac{d\sigma_{f,pasT}}{de_{f}}$. | (22) |
| --- | --- | --- |

#
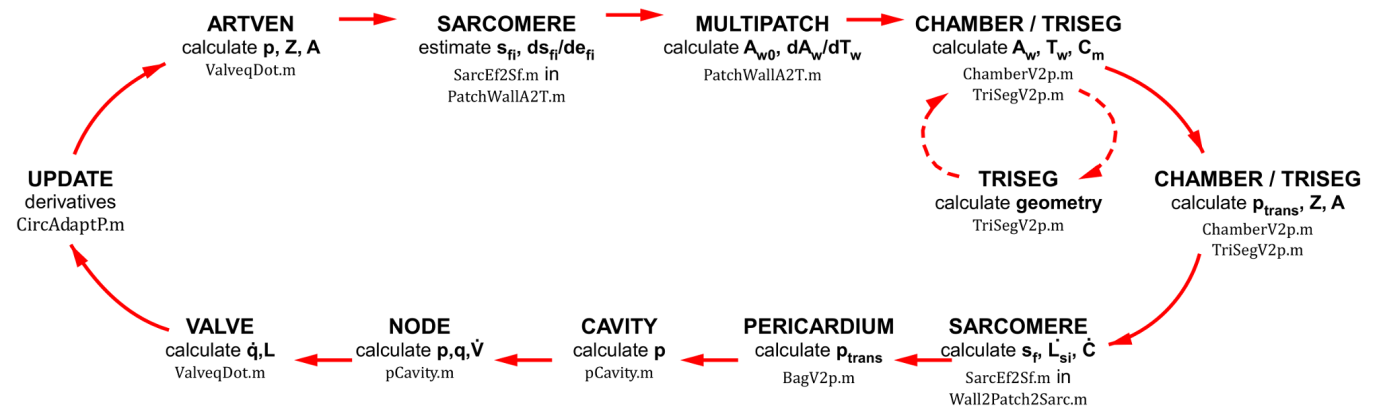


Figure 1 Calculation loop in the CircAdapt model

# Conservation of energy

CircAdapt connects wall tension T and wall area A to fibre stress σ_f_ and strain ε_f_ through the law of conservation of energy. Due to the transmural averaging assumptions in CircAdapt, changes in wall tension and area within a patch or wall must correspond to changes in fibre stress and strain throughout the volume of that patch or wall,

|  | $T dA= V_{w} \sigma_{f} d\varepsilon_{f}$ | (23) |
| --- | --- | --- |

Hence,

|  | $T=V_{w} \sigma_{f} \frac{d\varepsilon_{f}}{dA}$ | (24) |
| --- | --- | --- |

From the relation between fibre stress and wall area (Eq. 1 in main article), we have

|  | $\varepsilon_{f}=\frac{1}{2} \ln\left( \frac{A}{A_{Ref}} \right)$ | (25) |
| --- | --- | --- |

And so,

|  | $T=\frac{V_{w} \sigma_{f}}{2A}$ | (26) |
| --- | --- | --- |

# References

1. Lumens J, Delhaas T, Kirn B, Arts T (2009) Three-wall segment (TriSeg) model describing mechanics and hemodynamics of ventricular interaction. Ann Biomed Eng 37: 2234-2255.

2. de Tombe PP, ter Keurs HE (1990) Force and velocity of sarcomere shortening in trabeculae from rat heart. Effects of temperature. Circ Res 66: 1239–1254.

3. ter Keurs HE, Rijnsburger WH, van Heuningen R, Nagelsmit MJ (1980) Tension development and sarcomere length in rat cardiac trabeculae. Evidence of length-dependent activation. Circ Res 46: 703-714.
