# Supplementary material for: Fast Simulation of Mechanical Heterogeneity in the Electrically Asynchronous Heart Using the MultiPatch Module
Source: PLoS Comput Biol. 2015 Jul 23;11(7):e1004284. doi: 10.1371/journal.pcbi.1004284 (PMC4512705; doi:10.1371/journal.pcbi.1004284)
Supplement: S9 Text — Manual for using the enclosed CircAdapt model. (DOC) [file pcbi.1004284.s009.doc]

# The CircAdapt model

T. Arts, T. Delhaas, F. Prinzen, J. Lumens

Maastricht University, The Netherlands

The CircAdapt model is a lumped model of heart and circulation, simulating beat-to-beat hemodynamics and mechanics. The basis of the model has been presented earlier (*Arts T, Delhaas T, Bovendeerd P, Verbeek X and Prinzen FW. Adaptation to mechanical load determines shape and properties of heart and circulation, the CircAdapt model. Am J Physiol Heart Circ Physiol 288: 1943-1954, 2005; Lumens J, Delhaas T, Kirn B, Arts T (2009) Three-wall segment (TriSeg) model describing mechanics and hemodynamics of ventricular interaction. Ann Biomed Eng 37: 2234-2255*). The model is composed of a few types of elements, i.e. muscular chambers, elastic tubes, valves and peripheral resistances. Chambers are composed of sheets. The size of the constituting elements adapts to mechanical load, as generated by the model itself.

## Setup of the model

The model is schematically presented in Fig. (1).


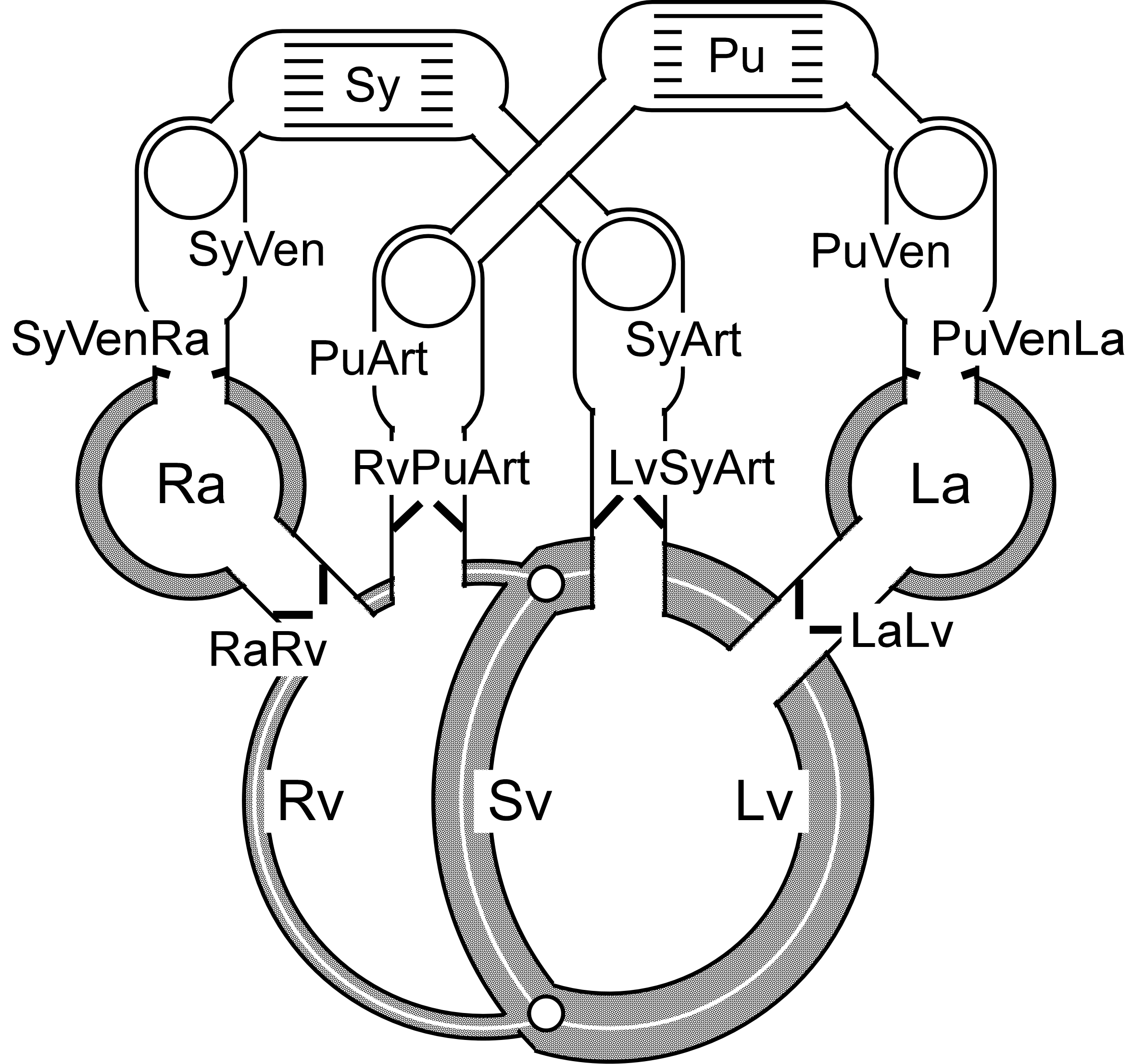


Fig 1: Schematic presentation of the CircAdapt model of the whole circulation. Meaning of the symbols: Sy, Pu= systemic and pulmonary circulation; Rv, Lv and Sv, = right and left ventricular cavities and free walls, and septal wall, respectively; Ra, La = right and left atrium; SyVenRa, RaRv, RvPuArt = Ra inflow channel, tricuspid valve and pulmonary valve; PuVenLa, LaLv, LvSyArt = La inflow channel, mitral valve and aortic valve; SyVen, PuArt, PuVen, SyArt = systemic veins, pulmonary artery, pulmonary veins and aorta.

The core of the model is a system of differential equations with a limited number (e.g. 30) of state variables (SVar). The set of state variables uniquely defines the state of the whole circulation. The set of time derivatives of the state variables is a unique function of the set of state variables, represented by the program part ‘SVarDot’. The system of differential equations has been solved in Matlab (function ‘ode113’) by scaling of the state variables to values around 1. All parameters and variables are stored in a comprehensive data structure ‘P’.

The Rv and Lv cavity are enclosed by three walls, i.e. Rv free wall, septal wall (Sv) and Lv free wall. The La and Ra cavities are enclosed by single walls La and Ra, respectively. Walls are composed of one or more patches. A patch has a mid-wall area and a thickness, equal to patch volume divided by patch mid-wall area. A patch contracts if the myofibers with sarcomeres contract. Entrances of valves and tubes are considered as non-contractile patches, encapsulating the cavities together with the contractile patches. Stress in the myofibers depends on strain and strain rate according to physiological experiments on isolated myofibers.

As seen from the heart, arterial inlets and venous outlets are bridged by ArtVen-elements, representing a circulatory section with peripheral resistance of the related micro-circulation. An ArtVen element consists of 5 elements, i.e. arterial and venous wave impedances and compliances, enclosing a peripheral resistance (*Arts T, Reesink K, Kroon W, Delhaas T (2011) Simulation of adaptation of blood vessel geometry to flow and pressure: Implications for arterio-venous impedance. Mech Re Commun In Press*). Venous inlets to the atria are similar to arterial outlets with the only difference the direction of average flow. So, changing pressures in the atria induce flow waves back into the veins.

Like in the real circulation, arterial blood pressure is controlled by variation of circulating blood volume.

## List of symbols and abbreviations

Elements and variables have been named carefully.

### Elements

An element is named by a concatenation of string fragments, each having the first letter capitalized. The structure as follows:

- ArtVen element: systemic and pulmonary (Sy, Pu), containing arteries (SyArt, PuArt) and veins (SyVen, PuVen).
- Heart with atria La, Ra and ventricles Lv, Rv
- Heart walls of atria La, Ra and ventricles with septum Lv, Rv, Sv
- Each wall has 1 or more patches, numbered successively, e.g., Lv1
- Each cavity has a node with the name of the cavity. Heart cavities La, Ra, Lv, Rv, and blood vessel cavities SyArt, PuArt, SyVen, PuVen.
- Valves are named after concatenation of the proximal and distal node, e.g. LvSyArt represents the aortic valve, LaRa represents ASD= atrial septal defect.

The following abbreviations for anatomical location have been used:

- Sy: systemic
- Pu: pulmonary
- Art: arterial
- Ven: venous
- a: atrium
- v: ventricle
- L: left
- S: septal
- R: right
- Peri: pericardium

### Variables

Variables are named, starting with their physical symbols, followed by further specifications. All variables are expressed in SI-units:

| ***Symbol*** | ***Unit*** | ***Description*** |
| --- | --- | --- |
| X, Y, Len | m | Length |
| A | m^2^ | Area |
| V | m^3^ | Volume |
| C | m^-1^ | Curvature= 1/radius |
| C | - | Time-variant contractility of sarcomere |
| p | Pa= Nm^-2^ | Pressure |
| q | m^3^s^-1^ | Flow |
| R | Pa m^-3^s | Hemodynamic resistance |
| Z | Pa m^-3^s | Hemodynamic impedance |
| L | Pa m^-3^s^2^ | Inertia |
| t, Tau, T | s | Time |
| T | Nm^-1^ | Wall tension |
| rhob | kg m^-3^ | Density of blood |
| Ls | micrometer | Sarcomere length (not SI!) |
| Ef | - | Strain of fibers |
| Sf | Pa= Nm^-2^ | Stress in fibers |
| v | ms^-1^ or micrometer/s | Velocity |
| *For handling of the differential equations:* | | |
| SVar | [-] | State variable (normalized by scaling) |
| <var>Dot | [-] | Time derivative of <var> |

Variables related to setpoints of adaptation are stored in P.ArtVen.Adapt and P.Patch.Adapt structures.

## The program

Properties of all elements are stored in tree-organized structure P, globally accessible throughout most of the program and functions. Properties of elements of the same type are stored in a single structure. Each column indicates a different element, each row indicates a new time sample. The names of the elements are stored in a row of strings within the same structure.

The 1^st^ level of P indicates the following sub-structures and numerical data

P =

General: [1x1 struct]: general data

ArtVen: [1x1 struct]: ArtVen elements, 2 cavities

Chamber: [1x1 struct]: Chamber elements, 1 cavity and 1 wall

TriSeg: [1x1 struct]: TriSeg element, 2 cavities and 3 walls

Cavity: [1x1 struct]: indexed by ArtVen, Chamber and TriSeg

Wall: [1x1 struct]: indexed by Chamber and TriSeg

Patch: [1x1 struct]: indexed by walls

Node: [1x1 struct]: connecting cavities and other elements

Bag: [1x1 struct]: passive eleastic bag like pericardium

Valve: [1x1 struct]: Valve, connecting nodes

t: [427x1 double]: time

Adapt: [1x1 struct]: general data used for adaptation

SVar: [853x30 double]: 853 time points of 30 state variables

tDot: [427x1 double]: derivative of time=1

SVarDot: [427x30 double]: derivative of SVar

>> P.General

rhob: 1050: density of blood

q0: 8.5000e-005: cardiac output

p0: 12200: mean aortic pressure

tCycle: 0.8500: cycle time

FacpControl: 1.0000: pressure control 0→no control, 1→control

ScaleVqY: [1e-005 1e-004 0.1]: scaling factors for SVar

Dt: 0.0020: time step

tEnd: 2.9750: end time of simulation

tCycleRest: 0.8500: cycle time at rest

TimeFac: 1: scaling for time compression of contraction

TauAv: 0.1573: AV delay in AV-node

DtSimulation: 1.2750: duration of simulation

>> P.ArtVen : ArtVen system

Name: {'Sy' 'Pu'}: systemic, pulmonary system

n: 2: number of Artven systems

iCavity: [1 3]: index of arterial cavity in P.Cavity

iWall: [1 3]: index of arterial wall in P.Wall

Adapt: [1x1 struct]: target values of adaptation

k: [2x2 double]: wall stiffness parameters

Len: [2x2 double]: length of arterial/venous tree

A0: [2x2 double]: reference cross-sectional areas

p0: [2x2 double]: reference pressures

AWall: [2x2 double]: cross-sectional wall areas

p0AV: [1.2093e+004 1500] pressure drop reference

q0AV: [8.5000e-005 8.5000e-005] flow reference

kAV: [1 2]: exponent non-linearity peripheral resistance

q: [427x2 double]: peripheral flow

>> P.Chamber,P

Name: {'La' 'Ra'}: left and right atrium

n: 2: number of chambers

iCavity: [5 6]: index to P.Cavity

iWall: [5 6]: index to P.Wall

>> P.TriSeg

Name: {'v'}: ventricles

n: 1: number of TriSegs

iCavity: 7: index to P.Cavity

iWall: 7: index to P.Wall

V: [427x1 double]: approximation VS, (→SVar)

Y: [427x1 double]: approximation YS, (→SVar)

Tau: 0.0050: time constant of V, Y solution

VS: [427x1 double]: volume shift septum

YS: [427x1 double]: radius of ventricular junction circle

VDot: [427x1 double]

YDot: [427x1 double]

>> P.Cavity

Name: {'SyArt' 'SyVen' 'PuArt' 'PuVen' 'La' 'Ra' 'Lv' 'Rv'}

n: 8: number of cavities

iNode: [1 2 3 4 5 6 7 8]: index to connected nodes

V: [427x8 double]: volume (→SVar)

Adapt: [1x1 struct]: structure of adaptation parameters

A: [427x8 double]: approximation cross-sectional area

Z: [427x8 double]: wave impedance between node and cavity

p: [427x8 double]: cavity pressure

VDot: [427x8 double]:

>> P.Wall

Name: {1x10 cell}: wall names

('SyArt' 'SyVen' 'PuArt' 'PuVen' 'La' 'Ra' 'Lv' 'Sv' 'Rv' 'Peri')

n: 10: number of walls

nPatch: [0 0 0 0 1 1 1 1 1 0]: number of patches per wall

iPatch: [1 1 1 1 1 2 3 4 5 6]: index first patch in P.Patch

AmDead: [0 0 0 0 13e-4 13e-4 13e-4 0 13e-4 0]: non-contractile area

Am0: [427x10 double]: zero tension mid wall area

DADT: [427x10 double]: dA/dT

T: [427x10 double]: wall tension

Cm: [427x10 double]: wall curvature

Am: [427x10 double]: mid-wall area

pTrans: [427x10 double]: transmuaral pressure

VWall: [1x10 double]: wall volume

>> P.Patch

Name: {'La1' 'Ra1' 'Lv1' 'Sv1' 'Rv1'}

n: 5: number of patches

SARCOMERE↓

Lsi: [427x5 double]: unloaded sarcomere length (→SVar)

C: [427x5 double]: contractile activation (→SVar)

ActivationDelay: [2x5 double]

LsRef: [2 2 2 2 2]

Ls0Pas: [1.8000 1.8000 1.8000 1.8000 1.8000]

dLsPas: [0.6000 0.6000 0.6000 0.6000 0.6000]

SfPas: [1x5 double]

Lsi0Act: [1.5100 1.5100 1.5100 1.5100 1.5100]

LenSeriesElement: [0.0400 0.0400 0.0400 0.0400 0.0400]

SfAct: [84000 84000 120000 120000 120000]

vMax: [14 14 7 7 7]

TimeAct: [0.1500 0.1500 0.4250 0.4250 0.4250]

TR: [0.4000 0.4000 0.2500 0.2500 0.2500]

TD: [0.4000 0.4000 0.2500 0.2500 0.2500]

CRest: [0 0 0 0 0]

SARCOMERE↑

VWall: [1x5 double]: wall volume

AmRef: [0.0068 0.0059 0.0096 0.0048 0.0125]: ref. area

Adapt: [1x1 struct]: adaptation target values

T: [427x5 double]: wall tension

Ef: [427x5 double]: fiber strain

Ls: [427x5 double]: sarcomere length

CDot: [427x5 double]:

SfEcm: [427x5 double]: ECM stress

SfPasT: [427x5 double]: total passive stress

LsiDot: [427x5 double]:

Sf: [427x5 double]: myofiber stress

DSfDEf: [427x5 double]: dSf/dEf

DADT: [427x5 double]: d(wall area)/d tension

Am0: [427x5 double]: zero stress wall area

Am: [427x5 double]: mid-wall area

>> P.Node: nodes connect elements

Name: {'SyArt' 'SyVen' 'PuArt' 'PuVen' 'La' 'Ra' 'Lv' 'Rv'}

n: 8

iCavity: [1 2 3 4 5 6 7 8]: indices referring to primary cavity

q: [427x8 double]: total inflow, should =0

Y: [427x8 double]: total conductivity

p: [427x8 double]: node pressure

>> P.Bag: passive elastig bag, like pericardium

Name: {'Peri'}

n: 1

iWall: 10: index referring to wall

VRef: 6.7167e-004: reference volume

k: 10: stiffness parameter

pAdapt: 100: reference pressure in Pa

p: [427x1 double]: actual pressure

>> P.Valve

Name: {1x9 cell}

('SyVenRa' 'RaRv' 'RvPuArt' 'PuVenLa' 'LaLv' 'LvSyArt' 'LaRa' 'LvRv' 'SyArtPuArt')

n: 9

iNodeProx: [2 6 8 4 5 7 5 7 1]: index of proximal node

iNodeDist: [6 8 3 5 7 1 6 8 3]: index of distal node

q: [427x9 double]: flow (→SVar)

AOpen: [1x9 double]: forward cross-section

ALeak: [1x9 double]: leak cross-section

Len: [1x9 double]: channel length

L: [427x9 double]: inertia

qDot: [427x9 double]

>> P.Adapt : records needed for definition of adaptation type

FunctionName: 'Adapt0P': used adaptation file

Fast: 0: [1,0] implies [fast,slow] steady state

In: [1x8 double]

Out: [1x8 double]

FlowVec: [1x9 double]

>> P.ArtVen.Adapt: adaptation target values blood vessels

WallStress: [2x2 double]: wall stress

vFlowMean: [2x2 double]: mean flow velocity

vImpact: [2x2 double]: body impact velocity

>> P.Patch.Adapt: adaptation target values sarcomere

LsBe: [2.3000 2.3000 2.3000 2.3000 2.3000]: begin ejection Ls

LsEe: [1.7500 1.7500 1.7500 1.7500 1.7500]: end ejection Ls

SfPasMax: [60000 60000 6000 6000 6000]: max ECM stress

SfPasAct: [6800 6800 4800 4800 4800]: integrine related stress

FacSfAct: [0.3500 0.3500 0.6100 0.6100 0.6100]: fraction of SfAct

The program uses the P-structure, stored in file ‘P.mat’ in the working directory. During execution of the program, P is globally accessible, allowing derivation of information everywhere in the program about any location in the circulation.

### Using the program

### Introduction

For a simple start do the following.

Store all program files (*.m-files) in a program directory (e.g. called ProgDir).

Start up MatLab.

Go to a working directory.

Open a path to the program directory by >>addpath ProgDir

Start the program by typing:

>> CircAdaptMainP <Enter>

[N]ew, [R]eference, [L]oad, [C]ontinue <Enter>: **r**

type: R (Reference) to load PRef.mat, representing a standard state of the circulation.

[P]ressure (kPa): 12.2

[F]low (ml/s): 85

cycle [T]ime (ms): 850

[D]uration simulation (s): 1.275

Adapt n[O]ne [R]est,[E]xercise : Adapt0

Faster steady state [Y]/[N] : N

<Enter> = Continue

Choose Letter <Enter>:

If nothing needs to be changed (default), press <Enter>

t= 1.7; Time to go= 1.275

Flow/q0: SyVenRa RaRv RvPuArt PuVenLa LaLv LvSyArt

1.0001 0.9981 0.9954 1.0001 0.9955 0.9953

Relative FlowError 1000x= 499

t= 2.55; Time to go= 0.425

Flow/q0: SyVenRa RaRv RvPuArt PuVenLa LaLv LvSyArt

1.0000 0.9978 0.9953 1.0001 0.9956 0.9954

Relative FlowError 1000x= 0

Differential equation has been solved

After each simulated beat, mean flow through 6 cardiac valves are printed out to judge the occurrence of steady state. (atrial inflows are handled as valves.) Time ‘t’ increases, while the ‘time to go’ decreases until the end of the simulation.

The model will simulate 2 beats (default). A selection of results is shown graphically in figure 1. All units are shown in SI-units. Calibration factors are shown at the top of the figure.

Upper left pane: PV-loops of Lv, Rv, La and Ra. Upper mid pane: stress(strain) plot of myofibers. Zero strain refers to sarcomere length 2um. Lower left pane: diastolic pressures in Lv, Rv, La, Ra, PuVen, SyVen and pericardium. Right pane: top tracings refer to left hemodynamics, i.e. pressures and volumes of Lv,La, aortic pressure, venous inflow, mitral flow and aortic flow. At lower left, data are similar, but now derived from the right side.

### Intervention by changing parameter setting

Any parameter in the circulation can be changed. After changing a parameter setting in the structure Par, this structure should be stored as file ‘P.mat’, because each simulation starts from reading this file. Below an example has been shown to simulate aortic valve stenosis.

Execute a normal beat by <CircAdaptMain> and <Enter>’s. Now, structure ‘P’ is in memory. Make a change to aortic valve stenosis by

Watch current cross-sectional area of open aortic valve

>> Aux= GetFt('Valve','AOpen','LvSyArt')

Aux = 4.9721e-004

Set new value of open artic valve cross-section

>> PutFt('Valve','AOpen','LvSyArt',0.2*Aux)

>> save P P

Increase simulation time to 10s as follows:

>> CircAdaptMainP **<Enter>**

[N]ew, [R]eference, [L]oad, [C]ontinue <Enter>: **<Enter>**

[P]ressure (kPa): 12.2

[F]low (ml/s): 85

cycle [T]ime (ms): 850

[D]uration simulation (s): 1.275

Adapt n[O]ne [R]est,[E]xercise : Adapt0

Faster steady state [Y]/[N] : N

<Enter> = Continue

Choose Letter <Enter>: **d**

Duration of simulation (s): **10**

Similarly, starting time may be set to zero by:

>> P.t(end)=0;

>> save P P

### Starting from New, and using adaptation

The reference file has been obtained by starting from scratch, followed by adaptation of the myocardium to mechanical load under hemodynamic exercise conditions. Next vessel diameters will be adapted to flow under resting conditions. This procedure is repeated a few times, leading to the reference ‘P’-structure, which is stored as file PRef.mat. Note that the scratch state at the beginning is not influencing the final result. However, the scratch state should be in a range that the solution is stable, allowing convergence to a stable physiologic state.

>> CircAdaptMainP

[N]ew, [R]eference, [L]oad, [C]ontinue <Enter>: **n**

Start with simulation from scratch by typing 'n' .

[P]ressure (kPa): 12.2

[F]low (ml/s): 85

[T]ime of beat (ms): 850

[D]uration simulation (s): 1.275

Adapt n[O]ne [R]est,[E]xercise : Adapt0P

Faster steady state [Y]/[N] : N

<Enter> = Continue

Choose Letter <Enter>: < type Enter >

t= 0; Time to go= 1.275

Flow/q0: SyVenRa RaRv RvPuArt PuVenLa LaLv LvSyArt

0.7973 0.8922 0.7830 0.8529 0.8777 0.9213

Relative FlowError 1000x= 485

t= 0.85; Time to go= 0.425

Flow/q0: SyVenRa RaRv RvPuArt PuVenLa LaLv LvSyArt

0.9739 0.9666 1.0023 0.9771 0.9632 0.9525

Relative FlowError 1000x= 85

Differential equation has been solved

Start with simulation from scratch by typing N.

Adapt vascular properties by adaptation at rest:

>> CircAdaptMainP **<Enter>**

...

[P]ressure (kPa): 12.2

[F]low (ml/s): 85

[T]ime of beat (ms): 850

[D]uration simulation (s): 42.5

Adapt n[O]ne [R]est,[E]xercise : Adapt0P

Faster steady state [Y]/[N] : N

<Enter> = Continue

Choose Letter <Enter>: **r**

Now the menu shows the change in adaptation property.

...

Adapt n[O]ne [R]est,[E]xercise : **AdaptRest**

Press <Enter>. Wait for many beats, while adaptation takes place.

Now bring to exercise by 3-fold increase of blood flow, 50% reduction of cycle time. Simulate with ‘Faster steady state’ option or set duration to 20 s.

>> CircAdaptMain <Enter>

[P]ressure (kPa): 12.2

[F]low (ml/s): **255**

cycle [T]ime (ms): **425**

[D]uration simulation (s): 1.275

Adapt n[O]ne [R]est,[E]xercise : Adapt0

Faster steady state [Y]/[N] : **Y**

<Enter> = Continue

Choose Letter <Enter>:

Adapt to Exercise condition:

>> CircAdaptMain

[N]ew, [R]eference, [L]oad, [C]ontinue <Enter>:

[P]ressure (kPa): 12.2

[F]low (ml/s): 255

cycle [T]ime (ms): 425

[D]uration simulation (s): **42.6**

Adapt n[O]ne [R]est,[E]xercise : **AdaptExc**

Faster steady state [Y]/[N] : N

<Enter> = Continue

Choose Letter <Enter>: **<Enter>**

After many beats, convergence occurs. If convergence is not yet complete, repeat an adaptation session to exercise.

Bring back to rest condition by reducing flow and heart rate to default values. Simulate with ‘Faster steady state’ option to reach steady state.

Use now ‘Adapt to Resting condition’.

…

[F]low (ml/s): **85**

cycle [T]ime (ms): **850**

…

Adapt n[O]ne [R]est,[E]xercise : **AdaptRest**

…

Wait for many beats, while adaptation takes place. The thus found state approximates the reference state sufficiently accurately for most applications. Accuracy may be increased by repeat of exercise and rest adaptation.

### Storing and retrieving a simulation

The reference simulation is stored as PRef.mat, obtained by storing structure P:

>>save PRef P

On may use any name for a mat-file, containing structure P. The simulation may be retrieved by

>> CircAdaptMain, followed by selecting (**L**)oad.

The most compact way of storing a simulation is storage of the start condition only by execution of

SVarDot(0,P.SVar(end,:)',[ ]), save Pxxx P

The most complete simulation with all time tracings is obtained by executing CircAdaptMainP, and storing P as a file. By retrieving this file, all time courses are ready for graphical display, e.g., aortic flow velocity is plotted as a function of time:

>> load Pxxx (mat-file containing P)

>> figure(2); plot( P.t, GetFt('Valve','q','LvSyArt')/... GetFt('Valve','AOpen','LvSyArt'));

## Program in parts

The program is composed of a number of m-files, which will be briefly described below. Structure Par is globally accessible throughout most of the function in the program.

### CircAdaptMainP

Script of main program to perform a CircAdapt simulation. Provides simple user interface.

### PNew

Creates a P-structure from scratch. Useful simulations require additional adaptation operations.

### CircAdaptP

The function CircAdaptP calculates a simulation, based on initial conditions, as defined by state variables in structure ‘P’. Many general conditions of the simulation are stored in ‘P.General’. Execution occurs in steps of whole cycles.

**GetFt('Cavity', 'p', 'Lv')**

Gets 'p'ressure in 'Lv'-'Cavity'

**PutFt('Cavity', 'p', 'Lv', 11000)**

Puts value of 11000 in P.Cavity.p(:,index of 'Lv')

### TimingP

The function Timing determines the sequence of activation of all patches.

### SVarDot

SVarDot calculates the set of time derivatives of state variables as a function of the set of state variables. To be used inside CircAdapt in combination with the Matlab ODE-solver.

Within SVarDot the following functions are used:

**SVar2P**; state variables SVar → physiologic representation P.xx

**MemAlloc:** necessary memory allocations

**ArtVenV2p:** volume → transmural pressure arteries and veins

**PatchWallA2T:** patch and wall linear function: Area Am= Am0+T*DADT

**ChamberV2p:** chamber volume → transmural pressure

**TriSegV2p:** TriSeg volumes → transmural pressures → Am, T

**Wall2Patch2Sarc:** Wall → Patch → Sarcomere LsiDot,CDot

**BagV2p:** Transmural pressure pTrans of elastic bag like pericardium

**pCavity:** Adding transmural pressures to absolute pressures

**pNodeVDot:** Node pressures and cavity VDot's

**ValveqDot:** Valve flow derivatives qDot

**P2SVarDot**; physiologic representation P.xx → d/dt state variables SVarDot

**SarcEf2Sf**

Sarcomere mechanics simulates the time dependent mechanical behavior of myocardial fiber. Used within function PatchWallA2T.

### CircDisplayP

Displays results of a simulation with state variables stored in ‘P.SVar’. All signals are derived from available state variables, and stored in ‘Par’, having its largest size. Several hemodynamic variables are shown graphically in a figure.

### Adapt0P

Pressure control between regular beats

### AdaptRestP

Between beats, a selection of adaptation actions is performed for the resting state (diameter of blood vessels) .

### AdaptExcP

Between beats, a selection of adaptation actions is performed for the state of exercise. (wall thickness of blood vessels and sheet geometry)

### PatchAdapt

Adaptation of patch wall thickness, ECM area and MyoM area to mechanical load

### ArtVenAdapt

Adaptation of diameter and wallthickness of arteries and veins

### P2SVar

Physiologic representation P.xx → state variables P.SVar

### Par2P

Conversion of Par-structure (previous CircAdap version) to P-structure

### SplitMerge

Splits or merges patches, which are part of a wall.

### SteadyStateP

Used to reach steady state faster (option 'Y').

### SparseDiag

Auxilary function to create sparse diagonal matrix.

### MapStructure

Maps a structure, presenting the tree of a structure as text.

### .mat files

- P.mat contains the state variables of the last simulation. For full availability of all variables within the P-structure, CircDisplayP has to be executed.

- PRef.mat contains starting conditions of the reference simulation.

- PExc.mat contains the start of the simulation of exercise.

- PTemp.mat contains all data of the last successfully executed beat in a series of beats, to be carried out within function ‘CircAdaptP’. The file is very useful for debugging purposes.
